# Cooling‐Induced Order–Disorder Phase Transition in CsPbBr_3_ Nanocrystal Superlattices

**DOI:** 10.1002/adma.202410949

**Published:** 2024-11-20

**Authors:** Umberto Filippi, Stefano Toso, Matteo L. Zaffalon, Andrea Pianetti, Zhanzhao Li, Sergio Marras, Luca Goldoni, Francesco Meinardi, Sergio Brovelli, Dmitry Baranov, Liberato Manna

**Affiliations:** ^1^ Istituto Italiano di Tecnologia Via Morego 30 Genova 16136 Italy; ^2^ International Doctoral Program in Science Università Cattolica del Sacro Cuore Brescia 25121 Italy; ^3^ Department of Materials Science University of Milano‐Bicocca Via R. Cozzi 55 Milano 20125 Italy; ^4^ Center for Nano Science and Technology Istituto Italiano di Tecnologia via Rubattino 81 Milano 20134 Italy; ^5^ Division of Chemical Physics and NanoLund Department of Chemistry Lund University P.O. Box, 124 Lund SE‐221 00 Sweden

**Keywords:** collective phenomena, nanocrystal superlattices, order–disorder, perovskite, phase transition, X‐ray diffraction

## Abstract

Perovskite nanocrystal superlattices are being actively studied after reports have emerged on collective excitonic properties at cryogenic temperatures, where energetic disorder is minimized due to the frozen lattice vibrations. However, an important issue related to structural disorder of superlattices at low temperatures has received little attention to date. In this work, it is shown that CsPbBr_3_ nanocrystal superlattices undergo a reversible order–disorder transition upon cooling to 90 K. The transition consists of the loss of structural coherence, that is, increased nanocrystal misalignment, and contraction of the superlattices, as revealed by temperature‐dependent X‐ray diffraction, and is ascribed to the solidification of ligands (on the basis of Raman spectroscopy). Introducing shorter amines on the nanocrystal surface allows to mitigate these changes, improve order, and shorten interparticle distance. It is demonstrated that the low temperature phase of the short ligand‐capped nanocrystal superlattices is characterized by a strong exciton migration observable in the photoluminescence decay, which is due to the shrinkage of the inter‐nanocrystal distance.

## Graphical abstract

This work reports a temperature‐driven order–disorder phase transition occurring in CsPbBr_3_ nanocrystal superlattices. Temperature‐dependent X‐ray diffraction and Raman spectroscopy reveal the loss of crystallinity of superlattices upon cooling and identify ligands solidification as the main driving force for this transition. Surface ligand engineering mitigates the structural degradation and results in strong exciton migration within the superlattice at cryogenic temperatures.

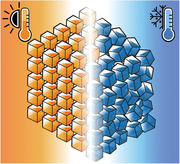

## 1 Introduction

Colloidal superlattices are highly ordered assemblies of nanocrystals separated by layers of surface‐bound ligands and held together by weak forces and hard‐particle interactions.^[^ ^1^, ^2^, ^3^ ^]^ The interest in these materials is fueled by their ability to display unique collective properties, not belonging to their individual building blocks: electronic coupling,^[^ ^4^ ^]^ plasmon‐polariton coupling,^[^ ^5^ ^]^ and magnetization alignment^[^ ^6^ ^]^ are just some examples of the intriguing behaviors displayed by superlattices of semiconducting, metallic, and magnetic particles, respectively. More recently, studies on superlattices of lead halide perovskites (CsPbX_3_, where X = Cl, Br, I) have drawn attention to excitonic coupling, which in these materials results in an unusually high exciton diffusivity^[^ ^7^ ^]^ and in quantum coherent light emission known as superfluorescence.^[^ ^8^ ^]^ Both phenomena are reportedly mediated by dipole–dipole coupling,^[^ ^9^, ^10^, ^11^ ^]^ which depends on the relative spatial position and exciton energy of the interacting particles. This makes CsPbX_3_ nanocrystals the ideal test system: their narrow size distribution leads to high energetic homogeneity,^[^ ^12^, ^13^ ^]^ while their faceted shape promotes higher order packing than spherical quantum dots,^[^ ^14^ ^]^ leading to a structural coherence comparable to that of epitaxial films.^[^ ^15^ ^]^

As the energetic and structural disorder are expected to affect the coupling strength, the optical properties of superlattices are often studied under cryogenic conditions, where the thermal quenching of lattice vibrations further narrows the emission spectrum and minimizes thermal decoherence.^[^ ^16^, ^17^, ^18^ ^]^ However, this implies the assumption that the structure of superlattices remains unchanged upon cooling, or at least that modifications are negligible. This is a reasonable expectation, since disorder is often associated with an increase in temperature and a superlattice contraction occurs upon cooling.^[^ ^19^ ^]^ Indeed, the few reported low‐temperature transformations in superlattices are phase transitions between different packing symmetries (for example, a Face‐Centered Cubic → Body‐Centered Tetragonal phase transition for spherical particles).^[^ ^20^, ^21^ ^]^ Hence, a significant phase transition is not expected in a simple‐cubic superlattice made of cuboidal CsPbX_3_ nanocrystals.

**Figure 1:**
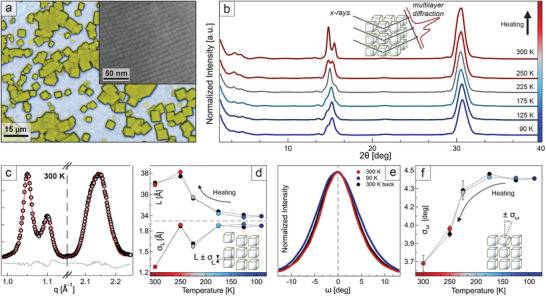
Cooling‐induced disorder in state‐of‐the‐art superlattices. a) Superlattices of CsPbBr_3_ nanocrystals capped with oleylamine and oleic acid (inset: high‐resolution scanning electron microscopy image of a superlattice surface). b) θ:2θ X‐ray diffraction patterns of the sample shown in panel (a) upon cooling (colored traces) and re‐heating (black overlayed traces) under vacuum. c) Example of multilayer diffraction fit of the two Bragg peaks, performed on the room‐temperature pattern from panel (b) (black = data, red = fit, gray = residual). d) Thermal evolution of the nanocrystals average misplacement parameter (σ_L_, bottom) and of the inter‐nanocrystals distance (L, top), extracted by fitting, as shown in panel (c), all the patterns in panel (b). e) Rocking curves collected at room temperature (red and black), and at 90 K (blue). f) Average nanocrystals angular distribution (σ_ω_), extracted from the rocking curves in Figure S4 (Supporting Information).

In this work, we demonstrate that the same CsPbBr_3_ superlattices that were subject to prior spectroscopy studies of cooperative effects^[^ ^8^, ^22^, ^23^, ^24^, ^25^, ^26^ ^]^ undergo an order–disorder phase transition upon cooling (**Figure** **1**), resulting in a substantial loss of crystallinity and decrease in interparticle distance. The transition is fully reversible for superlattices of CsPbBr_3_ nanocrystals capped with oleylamine and oleic acid and leaves no trace when the sample is brought back to room temperature, making it especially elusive. By means of Raman spectroscopy, we identify the solidification of ligands as the driving force for the cooling‐induced loss of structural coherence. In the attempt to counteract the loss in crystallinity of the superlattices at low temperature we engineered the surface passivation of nanocrystals by complementing oleic acid (always present) with shorter saturated amines. This modified ligand shell led to a higher degree of order and shorter interparticle separation both at room and cryogenic temperatures, although the decrease of structural coherence could not be fully suppressed and the reversibility of the transition was partially lost. We show that, for the superlattices at cryogenic temperatures, in correspondence with the low temperature phase where the inter‐nanocrystal distance reaches its minimum and phonon vibrations are suppressed, spectral diffusion compatible with a strong exciton migration is observable in the photoluminescence decay. In addition, a second, a redshifted peak appears, which can be attributed either to the transition dipole coupling of nanocrystals excited by exciton migration or to a subpopulation of larger nanocrystals.

## 2 State of Art Superlattices

The initial motivation of our study was to assess whether cryogenic temperatures could cause structural modifications in perovskite superlattices, as this might have an impact when investigating the collective properties of these materials. Since inter‐nanocrystal coupling typically encompasses very short ranges (≈10–100 nm), our analysis must be able to capture structural changes at comparable scales. Hence, we used multilayer diffraction, a method originally developed for epitaxial films that we recently repurposed to investigate the local structure of highly ordered nanocrystal solids.^[^ ^15^, ^27^, ^28^ ^]^ This approach is based on the analysis of fringes formed by the interference of X‐rays scattered by neighboring nanocrystals, which makes it ideal for tracking fine changes in parameters such as the interparticle distance and disorder at very local scales (< 100 nm).^[^ ^29^ ^]^

As a reference system, we selected superlattices of CsPbBr_3_ nanocrystals capped with oleylamine and oleic acid (Figure 1a). This is arguably the most common type of passivation for perovskite nanocrystals, and similar samples have been the subject of many spectroscopy studies.^[^ ^8^, ^22^, ^23^, ^24^, ^25^, ^26^ ^]^ Figure 1b shows the evolution of X‐ray diffraction (XRD) patterns from room temperature to 90 K under vacuum, corresponding to the operational limit of our setup. Only the (100) and (200) Bragg peaks of CsPbBr_3_ are visible due to the preferential orientation of nanocrystals in the sample. At room temperature, the first peak displays a fine structure characterized by sharp fringes. They represent a typical signature of multilayer diffraction that indicates a high degree of structural coherence (that is, low disorder), and it would not be possible to observe them in a highly disordered film (see Figure S1, Supporting Information). However, upon cooling the superlattices in vacuum (hence mimicking the conditions found in most spectroscopy cryostats), these fringes broaden significantly until the peak profile finally stabilizes at 200K. Such evolution is fully reversible, as seen from data collected while heating the sample back to room temperature (black in Figure 1b).

The fading of multilayer diffraction fringes indicates a degradation of the superlattices structural coherence, which can be quantified by fitting the fine structure of the Bragg peaks (Figure 1c; see also Figure S3 and Table S2, Supporting Information). This allows to extract both the interparticle distance L and the local disorder parameter σ_L_ that describes the average misplacement of particles from their ideal position. Figure 1d shows the evolution of both parameters, revealing a contraction of L (36.9 Å → 33.9 Å), and a major increase of σ_L_ (1.28 → 1.88 Å) when the sample is cooled to 90 K. This coincides with an increase in the angular misalignment of nanocrystals from their ideal orientation (σ_ω_ = 3.7° → 4.4°), that can be quantified via rocking curves acquired along the second Bragg reflection (Figure 1e–f; see also Figures S2 and S4, Supporting Information).

Notably, all parameters stop changing below 175 K, and show no hysteresis when the sample is heated back to room temperature, indicating that equilibrium conditions have been achieved at all steps. This behavior is reminiscent of a second‐order phase transition where a continuous variation of the disorder parameter with temperature connects two states of different structural coherence. A similar effect was recently observed between room temperature and 178 K in films of perovskite nanoplatelets, again by multilayer diffraction.^[^ ^30^ ^]^ These findings suggest that fine changes in the local structural coherence of nanocrystals assemblies might be rather common but require sensitive techniques to be detected. Crucially, the full reversibility of this transformation makes it likely to go undetected during spectroscopic investigations, since control experiments performed before and after the cooling cycle would bear no trace of it. However, the affected parameters are relevant to all coupling phenomena: for example, a contraction in the interparticle distance is expected to enhance the Forster resonant energy transfer (FRET) responsible for exciton diffusivity, while tilting of nanocrystals might affect the dipole–dipole coupling that is responsible for superfluorescence.^[^ ^8^, ^25^, ^26^, ^31^, ^32^, ^33^ ^]^ This considered, understanding such transformation is of great importance in informing future studies on superlattices.

## 3 Origin of the Order–Disorder Phase Transition

As noted above, the loss of structural coherence does not occur at a specific temperature, but rather in a temperature range. This excludes the hypothesis of a crystal structure change in the inorganic cores: indeed, the lowest‐temperature phase transition for CsPbBr_3_ occurs at 361 K, where the structure evolves from orthorhombic Pnma to tetragonal P4/mbm.^[^ ^34^ ^]^ Moreover, we observe a non‐linear trend in the evolution of σ_L_ that experiences a spike at 250 K in correspondence with a local maximum in the thermal trend of L.^[^ ^21^ ^]^ This suggests an involvement of the organic ligands in between the nanocrystals. Previous studies on superlattices of PbS nanocrystals and Au clusters with similar ligands (i.e., species with oleyl‐ tails) have highlighted the presence of glassy transitions in the ligand shell. In the case of PbS superlattices, this resulted in a phase transition of the nanoparticles packing from Face‐Centered Cubic to Body‐Centered Tetragonal,^[^ ^20^, ^21^ ^]^ while in superlattices of Au clusters it caused an order–disorder phase transition similar to what we observed here.^[^ ^35^, ^36^ ^]^ Despite the different nature of the material we are investigating, these reports suggest that the ligands might be primarily responsible for the degradation of structural coherence.

**Figure 2:**
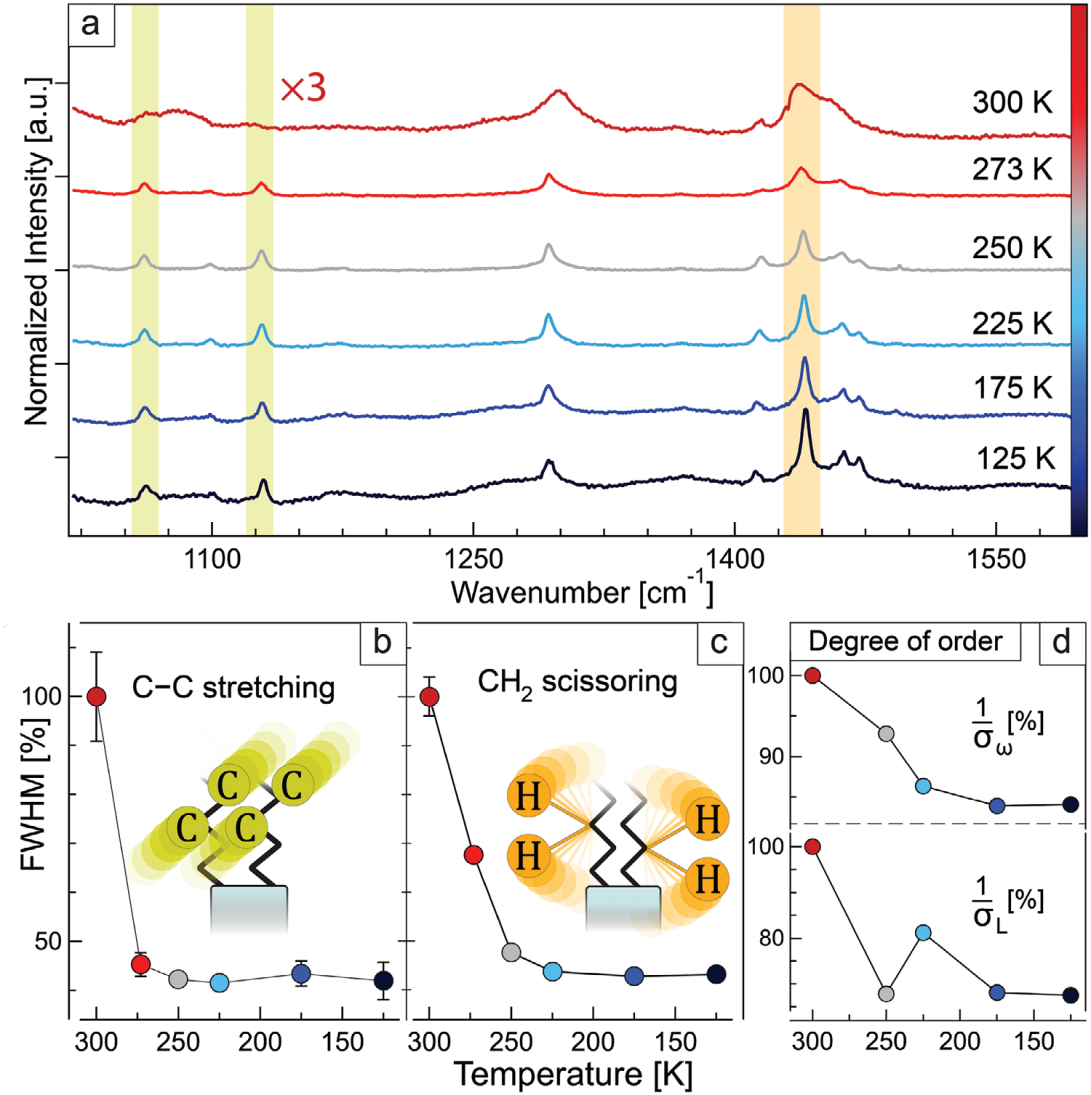
Raman spectra of ligands. a) temperature dependent Raman spectra (range 1020–1600 cm^−1^) of oleylamine/oleic acid coated nanocrystal superlattices. Full width at half maximum (FWHM) of the Raman peaks corresponding to: b) the C–C antisymmetric and symmetric stretching modes (1061 and 1120 cm^−1^ respectively) and c) CH_2_ scissoring modes (1437 cm^−1^). d) Degree of superlattice structural coherence expressed in percentage of 1/ σ_L_ and 1/σ_ω_.

To test our hypothesis, we studied the effect of temperature on the Raman signals of the ligands inside the superlattices. As seen in **Figure** **2**, most peaks become sharper as the sample cools down. For instance, tracking the signals attributed to the C–C antisymmetric and symmetric stretching modes (1061 and 1120 cm^−1^, respectively, Figure 2b) and to the CH_2_ scissoring mode (1437 cm^−1^, Figure 2c) allowed us to identify a sudden sharpening in the 300–225 K range, after which the peak widths remain substantially unchanged. This is consistent with a solidification of the ligands aliphatic chains: for reference, both oleic acid and oleylamine in pure form freeze at ≈280 K.^[^ ^37^, ^38^, ^39^ ^]^ Notably, such peak sharpening is concomitant with the loss of structural order, which we expressed in terms of 1/σ_L_ and 1/σ_ω_ in Figure 2d. This indicates the stiffening of ligands in between nanocrystals as the driving force for the observed order–disorder phase transition.

We hypothesize that the high temperature liquid‐like phase of the ligands bound to the surface of the nanocrystals allows the nanocrystals to adapt to the crystalline configuration of the superlattice, which is the one minimizing the energy of the assembly. At cryogenic temperatures, the thermal contraction of the inorganic and organic components of the superlattice would require nanocrystals to relocate to positions of new energy minima, resulting in a low temperature crystalline structure of the assembly. However, this step is precluded by the solidification of the ligand shell that kinetically locks the nanocrystals in their positions.

## 4 Minimizing Starting Disorder

**Figure 3:**
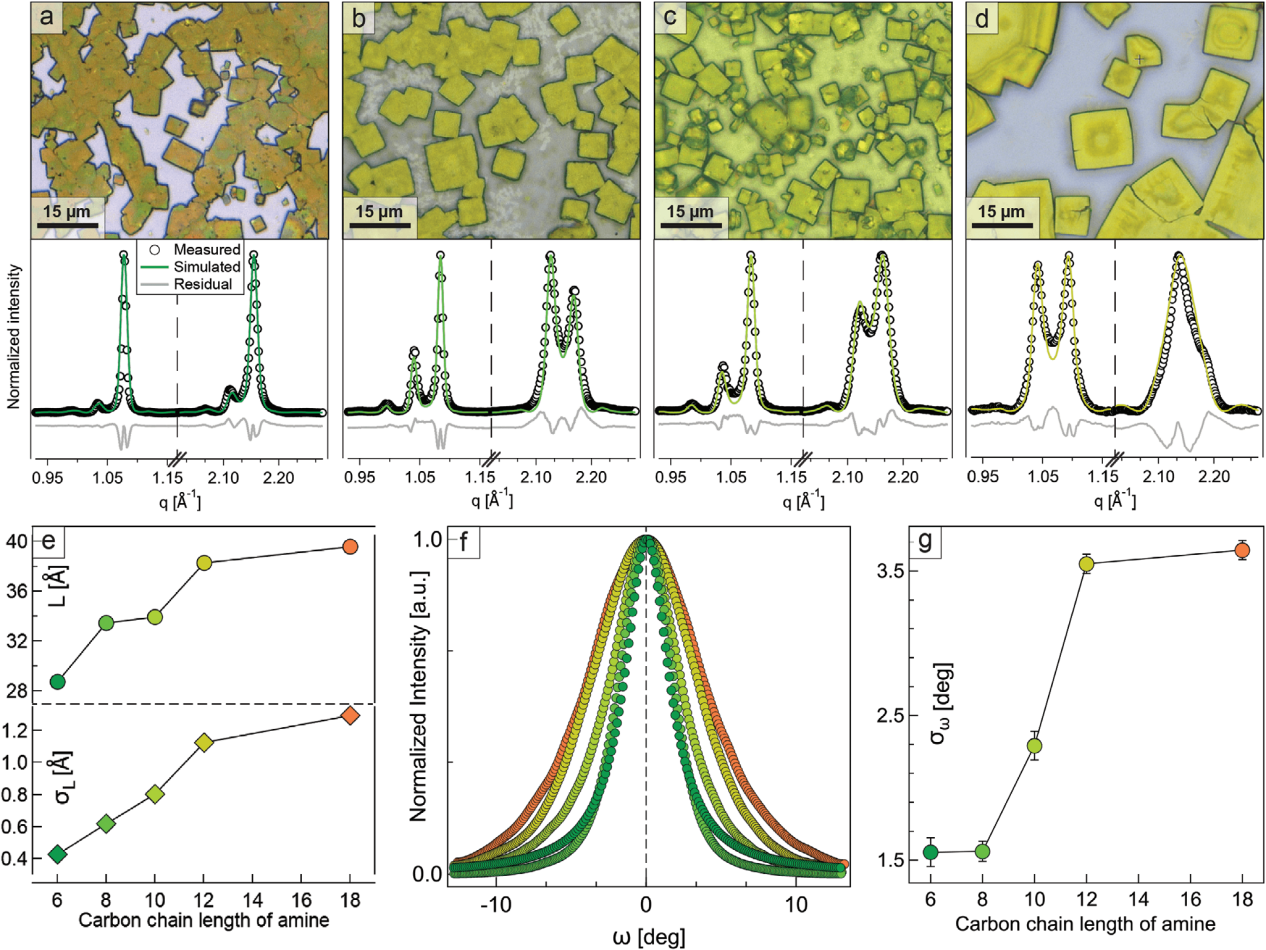
Superlattices with short‐chain amines. a–d) Top: optical microscopy images of superlattices prepared with short‐chain saturated amines. Bottom: corresponding multilayer diffraction fits of the Bragg peaks fine structures. From left to right: C_6_ (a), C_8_ (b), C_10_ (c), C_12_ (d). e) Correlations between the amine chain length (C_18_ = oleylamine) and the resulting superlattice structural parameters, as extracted from the multilayer diffraction fits: interparticle distance L and stacking disorder σ_L_, f) rocking curves for the second Bragg peak measured on superlattices prepared with different amines. g) Tilting disorder extracted by fitting the curves in (f).

As ligands appear to be at the origin of the order–disorder phase transition in CsPbBr_3_ superlattices, the best option to mitigate their effects is to engineer the surface of nanocrystals. Here, we opted to replace oleylamine with short‐chain saturated amines (hexyl‐ to dodecylamine [C_6_ – C_12_], see **Figure** **3**a–d). Such strategy was inspired by our previous studies on perovskite nanoplatelets, where the use of octylamine resulted in a significant reduction of both interparticle distance and stacking disorder^[^ ^27^ ^]^: we speculated that a better starting interdigitation of ligands and the absence of the stiff C═C group would help minimize the loss of structural coherence upon cooling. As expected, replacing oleylamine with shorter amines during the synthesis of nanocrystals (see Experimental Section) reduced the interparticle spacing in the resulting superlattices (Figure 3e, top), down to a minimum of L = 28.80 Å for hexylamine.

Likewise, both stacking (Figure 3e, bottom) and tilting disorder (Figure 3f,g) decreased significantly, again achieving their minima for hexylamine (σ_L_ = 0.42 Å, σ_ω_ = 1.56°, see Table S6 for multilayer diffraction and rocking curves complete fitting results). This is made evident by the multilayer diffraction fringes extending to the second Bragg peak of all samples, which is a clear signature of the enhanced structural coherence. However, the interparticle distances appear too long to match a double layer of the corresponding amines, and the discrepancy increases for shorter chains (e.g., L_C8_ = 33.5 Å vs expected 30.7 Å,^[^ ^40^, ^41^ ^]^ see, Table S7, Supporting Information). This suggests a mixed capping composed of both short amine and oleic acid, which was confirmed by nuclear magnetic resonance spectroscopy^[^ ^42^ ^]^ (NMR, see Figures S8–S11, Supporting Information).

This finding is consistent with previous surface studies on CsPbBr_3_ nanocrystals,^[^ ^43^, ^44^ ^]^ but marks an interesting difference with CsPbBr_3_ nanoplatelets stacks, where oleic acid is present in the synthesis but absent on the particles surface.^[^ ^27^ ^]^

**Figure 4:**
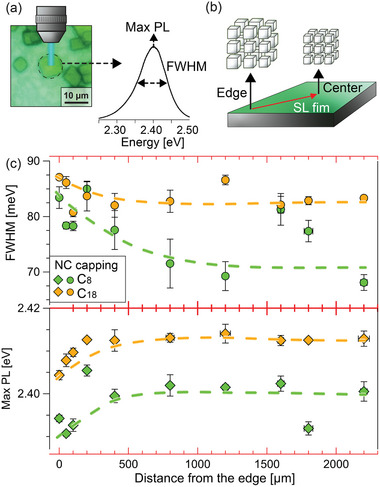
Spatial variability of superlattices PL across the sample. Micro‐PL data collected on individual oleylamine‐ and octylamine‐capped nanocrystal superlattices across a 1 cm × 1 cm silicon substrate. a) Microscopy image of a single superlattice, and scheme of the micro‐PL spectrum collection. b) Sketch of the superlattices distribution across the substrate: superlattices closer to the edges are mostly composed by larger and polydisperse nanocrystals, while those at the center tend to grow from smaller and more monodisperse particles. c) Average values PL FWHM (top) and peak position (bottom) of three superlattice sampled within a small region and plotted against the location of such region on the substrate.

Overall, we attribute the lower disorder displayed by short‐chain amine samples to several concurring effects. First, combining long oleic acid and short‐chain amine molecules leads to better interdigitation, which is likely facilitated by the intercalation of solvent, as discussed in the next paragraph. Second, shorter‐chain amines promote the growth of slightly larger nanocrystals (see Figures S12–S14, Supporting Information),^[^ ^45^ ^]^ which reduces the organic/inorganic fraction ratio and therefore decreases the superlattice “softness”.^[^ ^46^ ^]^ Finally, shorter‐chain amines appear to induce a stronger particle size selection during the self‐assembly of superlattices. The size selection was demonstrated by tracking the photoluminescence (PL) energy and spectral width of single superlattices in the sample from the edge to the center of the substrate (**Figure** **4**a). Upon visual observation, we noticed that superlattices grow first at the edges, and then the growth region progressively shifts toward the center. Since larger nanocrystals tend to precipitate first, this leads to macroscopic inhomogeneities: superlattices found at the center of the substrate, where we collected XRD data, tend to have higher PL energy (i.e., smaller particles) and a narrower PL width (i.e., narrower size distribution). Notably, the spatial variations of both parameters are more marked when a short amine, specifically octylamine, is used (light green markers, Figure 4b,c). This demonstrates that shorter amines induce a narrower particle size selection: however, it is unclear whether this is the cause of the improved structural coherence, or vice‐versa, it is a consequence of the nanocrystals being unable to join highly‐ordered superlattices formed by particles of different size.

## 5 Cooling C8‐Capped Superlattices

Based on Figure 3, hexylamine‐capped nanocrystals would be ideal to prepare highly ordered superlattices. However, we observed that the outcome of the assembly is less reproducible than for other amines, likely because these nanocrystals have a higher tendency to aggregate and fuse (see Figures S15 and S16, Supporting Information and related discussion). This motivated us to focus instead on samples prepared with octylamine (i.e., C_8_‐capped). Despite the excellent structural coherence (Figures S5–S7, Supporting Information), our initial attempts to cool C_8_‐capped superlattices under vacuum revealed a sudden and non‐recoverable disordering, with a complete loss of multilayer diffraction fringes already at 250 K (Figure S17, Supporting Information). However, control experiments showed that the cause was the vacuum itself: while oleylamine‐capped superlattices are almost unaffected, C_8_‐capped superlattices start degrading as soon as they become exposed to the vacuum (Figure S18, Supporting Information). Such degradation is characterized by a marked contraction of the interparticle distance (L ≈ 33.5 Å → 23.5 Å, see Figure S17, Supporting Information) and rapid disordering (σ_L_ ≈ 0.6 Å → 2 Å). These changes are due to the sudden evaporation of organic molecules originally located in between nanocrystals. These are likely solvent molecules and a fraction of the short‐chain amine molecules. This is confirmed by thermogravimetric analysis (Figure S18, Supporting Information), which showed that C_8_‐capped superlattices before and after vacuum treatment differ by a significant mass loss at ≈100 °C, amounting to 16% of the pre‐vacuum sample mass. This mass loss should also include excess organics non necessarily located in between the nanocrystals. This mass loss is then followed by a second loss (130—380 °C, 17%) attributed to less volatile and more strongly bound organic molecules, and by a final loss corresponding to the inorganic fraction of the superlattice (520 °C, 67%).

**Figure 5:**
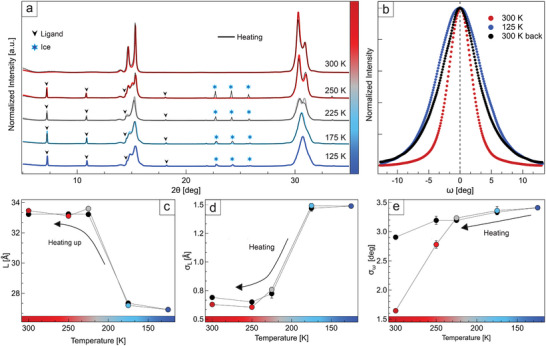
Low temperature behavior of octylamine‐coated nanocrystal superlattices: a) θ:2θ X‐ray diffraction patterns and b) rocking curves of octylamine‐coated nanocrystal superlattices measured for the second Bragg peak from 300 K to 125 K (dark red to dark blue traces) and back (black overlapping traces) in nitrogen atmosphere. The peaks belonging to ice flakes forming during the measurements and excess of ligands solidifying at low temperatures are marked with blue stars and black arrows respectively. Results of the multilayer diffraction and rocking curves fitting plotted as a function of temperature: c) interparticle separation, d) fluctuation of the interparticle separation and e) average nanocrystals angular distribution (σ_ω_).

To circumvent this issue of rapid disordering, we simply repeated the cooling experiment under a flow of N_2_, which however limited the operative range of our setup to 125 K (**Figure** **5**). This time, the sample retained a significant fraction of its initial structural coherence (σ_L_ = 0.63 → 1.49 Å), indicating that the stripping of organics was likely prevented. Notably, the interparticle distance still contracts significantly, reaching L ≈ 26.9 Å. This is 20% shorter than for oleylamine‐capped superlattices at the same temperature: considering that coupling interactions scale with 1/r^n^ (where n > 1 and depends on the specific interaction, e.g. n = 6 for FRET), this contraction might have major effects on the collective properties of superlattices. To rule out the possible impacts of the different experimental conditions, we performed temperature‐dependent XRD measurements on C_18_‐capped superlattices in N_2_ atmosphere and observed a comparable temperature change to that in vacuum (see Figure S19, Supporting Information). As for the oleylamine case, the evolution of both σ_L_ and L is fully reversible, again pointing to an order–disorder phase transition centered around ≈200 K. This was confirmed by Raman experiments performed on C_8_‐superlattices, which showed no significant differences with respect to oleylamine‐capped samples (see Figures S20 and S21, Supporting Information, Raman experiments were performed under N_2_ for both C_8_ and C_18_ samples). This is not surprising considering that the two ligand shells are rather similar, as they both contain a significant fraction of oleic acid. Nevertheless, the introduction of a shorter chain amine was still effective in retaining a much higher degree of structural coherence (for reference, at 125 K oleylamine yielded σ_L_ = 1.88 Å). To put these numbers in perspective, it is worth reminding that σ_L_ is the standard deviation of the nanocrystal misplacement. Therefore, the interpretation of such values should not follow a linear scale: while σ_L_ = 0 Å is unattainable perfection, and σ_L_ ≈ 0.1 Å is the typical displacement of atoms inside a highly crystalline solid, a value of σ_L_ = 10 Å = 1 nm would describe a group of loosely packed particles. Hence, it is evident how a 0.5 Å difference should not be underestimated when σ_L_ is in this range of values. Finally, we note that σ_ω_ does not recover fully (1.6° → 3.4° → 2.9°), even if it remains smaller than in the oleylamine case at all temperatures. However, we clarify that the tilting disorder is not measured locally, unlike σ_L_, because it is not extracted from the interference of X‐rays scattered by neighboring particles. As such, it can be affected by macroscopic tilts (e.g., that of entire superlattices) without the nanoscopic structure of the material being affected. Since the local stacking disorder σ_L_ is fully reversible, we conclude that σ_ω_ must also be locally reversible. We therefore attribute such drift to external factors: a possible reason is the crystallization of ice from moisture in the N_2_ flow (blue peaks), which might mechanically dislodge the superlattices from the substrate, leading to small residual tilts. Indeed, a control experiment performed by cooling and re‐heating the sample faster, to minimize the formation of ice, showed that, although the final low temperature values of σ_L_ and σ_ω_ are independent of the cooling rate, a much better reversibility is achieved (σ_ω_ = 1.6° → 3.4° → 2.0°, see Figures S22 and S23, Supporting Information).

## 6 Low Temperature Optical Properties

**Figure 6:**
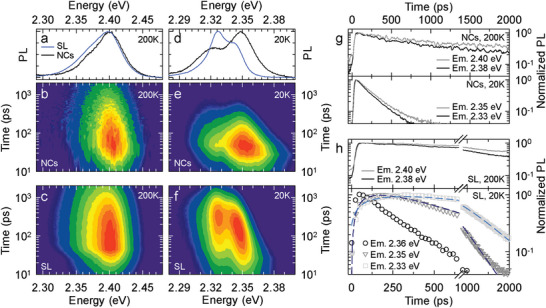
Low temperature optical properties: a) PL spectra of octylamine‐coated nanocrystals in form of isolated NCs dispersed in a polystyrene film (black line) and in form of assembled superlattices (blue line) measured at 200 K. The corresponding spectrally and time‐resolved PL decays are shown in b) and c), respectively. d–f) Same as “a‐c” at 20K. g) PL decay curve of the isolated octylamine‐coated NCs at 200 K (top panel) and at 20 K (bottom panel). h) Same as “g” for assembled superlattices. The dashed lines correspond to the PL decay fits using the decay dynamics of higher energy components as rise time.

Finally, motivated by the higher order and reduced interparticle spacing under cryogenic conditions, we investigated possible photophysical implications of the thermal structural evolution by temperature dependent PL measurements from 200 to 20K, where most phonon‐based processes are largely suppressed. Care was taken to work with freshly grown superlattices to avoid age‐related growth of bulk impurities,^[^ ^47^ ^]^ and to operate at low excitation fluence (≈3 nJ cm^−2^) in a helium atmosphere to avoid detrimental effects due to beam damage and vacuum‐induced disordering, respectively. **Figure** **6** shows the comparison of time‐resolved PL measurements on C_8_‐superlattices (indicated as “SL”) with similar measurements of C_8_‐nanocrystals dispersed in a polystyrene matrix (indicated as “NCs” in Figure 6 and as “isolated NC film” further in the text, prepared with a solution 27 times more diluted than the C_8_‐superlattices solution, see Experimental Section and Figure S24 (Supporting Information) for sample characterization). The time‐integrated PL spectra of the two samples at 200K are shown in Figure 6a together with the corresponding contour plots of the spectral and time‐resolved PL (Figure 6b,c). The PL spectra are essentially identical for both samples with a peak energy of 2.40 eV and uniform decay kinetics with no measurable rise time, indicating direct excitation by the 3.05 eV light and negligible interparticle spectral migration. Based on the spectral similarity, we attribute the PL to uncoupled nanocrystals. Upon cooling, however, the difference between superlattices and isolated NC film samples becomes striking (Figure 6d–f). The latter shows the expected redshift and narrowing of the PL peak (Figure 6d)^[^ ^48^, ^49^ ^]^ accompanied by the acceleration of the decay time due to the progressive bright triplet character of the exciton sub‐state responsible for the emission (Figure 6e,g).^[^ ^50^, ^51^ ^]^ Spectral diffusion is still absent, indicating the expected absence of interparticle interactions in the polymer matrix even at cryogenic temperatures. Notably, similar measurements on a film of nanocrystals spin‐cast onto a silica substrate without polystyrene yields a nearly identical spectral response at both 200 and 20 K (Figure S25, Supporting Information), indicating that also in the absence of intentional interparticle separation by the polymeric matrix, secondary interactions are negligible. A small low‐energy shoulder at 2.33 eV with identical lifetime as the main peak appears after direct excitation at low temperature, as shown by the identical signal rise time of the PL decay curves in Figure 6g. The possible origin of this contribution is discussed later in this section. The situation is markedly different in the superlattice, revealing enhanced coupling between the nanocrystals following thermal superlattice contraction. In particular, the time‐integrated PL is substantially narrower than the isolated NC film and is dominated by a low energy peak at ca. 2.33 eV (2.326 eV). The contour plot reveals the origin of the composite spectral shape with a high energy contribution undergoing ≈10 meV spectral diffusion from 2.35 to 2.34 eV in the first 250 ps. This is clear signature of exciton migration among a manifold of nearly resonant excited states due to a narrow size distributed population of nanocrystals in the superlattice. Accordingly, the PL at 2.35 eV grows slowly over time, with rise time coinciding with the PL decay of the high energy tail at 2.36 eV (Figure 6h and Figure S26). A further distinct low energy PL peak at 2.33 eV becomes clearly visible (Figure 6d,f), with an even slower rise time, now coinciding with the PL decay time at 2.35 eV, indicating that the main excitation source is a cascade of exciton migration processes inside the nanocrystal superlattice, further confirming the observation of a closer interparticle distance at cryogenic temperatures. Consistent with this picture, an analogous spectroscopic characterization of superlattices based on nanocrystals with longer C_18_ ligands yields an essentially intermediate behavior between the C_8_ nanocrystals superlattice and the corresponding disordered films (see Figure S27, Supporting Information), further suggesting a major role of the temperature‐dependent interparticle distance. The origin of the 2.33 eV contribution is however unclear. Following the findings of Rainò et al. on superfluorescent CsPbBr_3_ NC superlattices,^[^ ^8^ ^]^ one possible scenario could be cooperative emission by stronger dipole‐coupled NCs at low temperature, an interpretation that would be supported by the similar energy difference between two spectral contributions at 20 K and the reported literature case. The slow time dynamics observed in our systems with respect to the expected faster decay of the super‐radiant emission could be justified by the dominant indirect excitation by excitation migration leading to a PL decay similar to the kinetics of the migration process generating the coupling dipoles. However, the linewidth of the delayed low‐energy component is comparable to the high‐energy contribution, which is incompatible with the strong line narrowing expected for cooperative processes.

The low fluence regime used in our experiments might account for the absence of collective optical features. Indeed, when we excited our system at a temperature and fluence comparable to those of previous studies where superfluorescence was reported, we observed the appearance of redshifted and narrowed spectral feature with superlinear fluence dependence, partially compatible with previously reported signatures of superfluorescence (Figure S28, Supporting Information). On the other hand, the sequence of indirect excitation steps from progressively lower energy states shown in Figure 6h, without a direct connection between the high energy tail at 2.35 eV and the lowest delayed luminescence peak moving toward 2.33 eV, might suggest that the latter could be due to transition dipole coupling of slightly larger nanocrystals in the lattice following their excitation by exciton migration, somewhat similar to the delayed formation of excited state dimers in molecular solids. An alternative explanation could be the presence of a tiny but distinct subpopulation of slightly larger nanocrystals in the ensemble, which only become visible in the presence of strong interparticle energy migration causing their indirect predominant excitation. Consistent with this picture is the observation of the weak low‐energy shoulder in the isolated NC film, which may originate from an analogous but broader nanocrystal subpopulation after suppression of thermal quenching. The narrower spectral linewidth in the superlattice (Figure 6d) would also be consistent with nanocrystal size selection imposed by the assembly process. Similarly, we do not exclude that the delayed low‐energy PL could be due to indirect excitation of a small number of structural shallow defects, although this is less likely due to the observed fast emission lifetime, which is hardly compatible with localized trap states.

## 7 Conclusion

In summary, our experiments demonstrate that cryogenic conditions induce an order–disorder transition that degrades the structural order of CsPbBr_3_ nanocrystal superlattices. That transition is fully reversible and driven by the solidification and stiffening of the ligands between nanocrystals. The low‐temperature structural disorder leaves no trace when the sample is reheated, which makes it elusive to detect unless the structure is examined at low temperatures. The combination of multilayer diffraction and rocking curve experiments demonstrated here serves as a protocol to track the disorder and complement cryogenic optical studies of superlattices, as it could reveal transformations occurring in the nanocrystal assembly which are essential for data interpretation.

The ligands being a culprit of the transition, we demonstrated ligand engineering by replacing oleylamine with short‐chain amines (e.g., octylamine) in the synthesis of nanocrystals as a promising strategy toward the goal of obtaining cryo‐proof superlattices. The resulting oleate/short‐chain ammonium ligand shell facilitates the ligand interdigitation, decreases the superlattices softness, and significantly reduces effects of temperature‐induced disorder. The higher degree of structural and energetic homogeneity and the much shorter interparticle distance achieved in short‐amines CsPbBr_3_ superlattices and preserved in cryogenic conditions enabled observation of exciton migration between close packed and ordered nanocrystals possibly leading to coupling effects. The relative influences of the discovered structural changes in the superlattices upon cooling onto their optical properties remain to be studied.

## 8 Experimental Section

### Chemicals and Reagents

Lead(II)bromide (PbBr_2_, ≥98%), cesium carbonate (Cs_2_CO_3_, 99%), 1‐octadecene (technical grade, 90%), oleylamine (technical grade, 70%), oleic acid (technical grade, 90%), dodecylamine (technical grade, 98%), decylamine (technical grade, 99%), octylamine (technical grade, 99%), hexylamine (technical grade, 99%), toluene (anhydrous), ethyl acetate (≥99.5%), were purchased from Sigma–Aldrich and used without any further purification.

### Synthesis of CsPbBr_3_ Nanocrystals

CsPbBr_3_ nanocrystals were synthesized following previously reported method with minor variations.^[^ ^26^, ^28^ ^]^ In a 20 mL vial, 72 mg of lead (II) bromide (PbBr_2_, > 98%) were combined with 5 mL of ODE and different amount of amine and of oleic acid (OA), depending on the employed amine (see Table S1, Supporting Information). The temperature of the mixture was raised to 185 °C, then the vial was lifted from the block and fixed with a clamp above the hotplate and as soon as it cooled down to the injection temperature desired (160—175 °C depending on the amine employed, see Table S1, Supporting Information), 0.5 mL of the cesium oleate stock solution were swiftly injected (400 mg of Cs_2_CO_3_ dissolved in 1.75 mL of OA and 15 mL of ODE at 120 °C in nitrogen atmosphere). The reaction was quenched after 10 s with an ice–water bath under stirring. The crude solution was centrifuged for 6 min at 6000 rpm. For the synthesis employing oleylamine, the supernatant was discarded and the solution was centrifuged again at 4000 rpm for 3 min two times to discard any liquid. The remaining solid was dissolved in 300 µL of toluene, centrifuged and the supernatant was kept and stored for experiments. For the synthesis employing short‐chain amines (hexylamine, octylamine, decylamine, and dodecylamine), after the centrifugation of the crude solution, the supernatant was kept and 7 mL of ethyl acetate were added to it. The solution was centrifuged at 9000 rpm for 8 min. The supernatant was discarded and the solution was centrifuged again at 4000 rpm for 3 min to discard any remaining liquid. The remaining solid was dissolved in 300 µL of toluene, centrifuged and the supernatant was kept and stored for experiments.

### Preparation of Nanocrystal Superlattices

Self‐assembly of CsPbBr_3_ nanocrystals was performed by dropcasting 30 µL of solution of nanocrystals dispersed in toluene on silicon substrates placed inside a petri dish. The solvent was allowed to evaporate slowly (≈ 12 h) and after that the films were considered ready for experiments.

### Preparation of C_8_‐Nanocrystals Film

The disordered nanocrystal film was prepared by diluting the concentrated solution of C_8_‐capped nanocrystals used for superlattice preparation (concentration of dense solution ≈ 9.45 µm and of diluted solution ≈ 0.35 µm) in the presence of stabilizing polymer. Polystyrene was dissolved in toluene so to reach the 5% of weight solution, and 3 mL of this solution was mixed with 90 µL of the nanocrystal dense solution. 300 µL of the resulting mixture were spin coated at ≈ 1000 rpm on a 2 cm × 0.5 cm glass slide.

### Diffraction Data Collection

θ:2θ x‐ray diffraction patterns were acquired in Parallel Beam (PB) geometry, using a 3rd generation Malvern‐PANalytical Empyrean diffractometer equipped with a 1.8kW CuKα ceramic X‐ray tube (λ = 0.1541874 nm) operating at 45 kV and 40 mA, automated prefix iCore‐dCore optical modules for the incident and diffracted beam paths, PIXcel3D area detector and Anton Paar TTK 600 Low‐Temperature Chamber. Non‐ambient measurements were carried out on samples dropcasted on zero‐diffraction silicon substrate. Measurements were performed either in vacuum (2 × 10^−6^bar) or in dynamic nitrogen atmosphere.

### Morphology Characterization

The morphology of the sample was analyzed by using a field emission scanning electron microscopy (FE‐SEM) JEOL JSM‐7500 FA operating at 25 kV acceleration voltage and by a ZETA‐20 true color 3D optical profiler.

### Micro‐Raman and Micro‐PL Data Collection

Raman measurements were performed with a Raman spectrometer in Via Renishaw. For the measurements collected in the 1000–1800 cm^−1^ range, a 633 nm laser was used with 1.7 mW of power, equipped with a 1800 grooves/mm grating. Cryogenic experiments in the 300–90 K temperature range were performed with a THMS600 Linkam stage in nitrogen atmosphere. Room temperature micro‐PL measurements were performed with a 488 nm excitation laser with 5 × 10^−5^ mW of power with a 2400 grooves/mm grating. For detection, a ×50 long working distant microscope objective lens was employed.

### Cryogenic Photoluminescence and Time‐Resolved Measurements

The PL and time‐resolved PL (TRPL) experiments on the C_8_‐capped nanocrystal superlattices and isolated NCs were performed by using a frequency‐doubled Ti:sapphire laser (λ_exc_ = 400 nm, pulse duration = 150 fs, repetition rate = 78 MHz and beam diameter ≈ 200 µm) as excitation source and a Hamamatsu streak camera (time resolution ≈10 ps) as detector. To perform optical experiments at cryogenic temperatures, C_8_‐capped nanocrystals were placed in a in a closed‐cycle helium cryostat (Temperature range = 20–200 K) with a temperature controller with 0.001 K sensitivity and measured in helium atmosphere.

## Conflict of Interest

The authors declare no conflict of interest.

## Supporting information



Supporting Information

## Data Availability

The data that support the findings of this study are available from the corresponding author upon reasonable request. The experimental XRD data is available in the Zenodo repository.^[^ ^52^ ^]^
