# Supplementary material for: Cooling‐Induced Order–Disorder Phase Transition in CsPbBr3 Nanocrystal Superlattices
Source: Adv Mater. 2024 Nov 20;37(3):2410949. doi: 10.1002/adma.202410949 (PMC11756043; doi:10.1002/adma.202410949)
Supplement: Supplementary file 1 — Supporting Information [file ADMA-37-2410949-s001.docx]

*Supporting Information for*

**Cooling-Induced Order-Disorder Phase Transition**

**in CsPbBr_3_ Nanocrystal Superlattices**

*Umberto Filippi,^a,b^ Stefano Toso,^a^ Matteo L. Zaffalon,^c^ Andrea Pianetti,^c^ Zhanzhao Li,^a^ Sergio Marras,^a^ Luca Goldoni,^a^ Francesco Meinardi,^c^ Sergio Brovelli,^c*,^ Dmitry Baranov,^d,*^ Liberato Manna^a,*^*

*a) Istituto Italiano di Tecnologia, Via Morego 30, 16136 Genova, Italy*

*b) International Doctoral Program in Science, Università Cattolica del Sacro Cuore, 25121 Brescia, Italy*

*c) Department of Materials Science, University of Milano-Bicocca, Via R. Cozzi 55, 20125 Milano, Italy*

*d) Division of Chemical Physics and NanoLund, Department of Chemistry, Lund University, P.O. Box, 124, Lund, SE-221 00, Sweden*

*** [liberato.manna@iit.it](mailto:liberato.manna@iit.it) ; [dmitry.baranov@chemphys.lu.se](mailto:dmitry.baranov@chemphys.lu.se) ; [sergio.brovelli@unimib.it](mailto:sergio.brovelli@unimib.it)

# 1) Synthesis of CsPbBr_3_ nanocrystals.

**Table S1. Synthesis conditions.** Nature and amounts of surfactants, and injection temperatures employed for the synthesis of nanocrystals with different amine capping agents.

| Amine | Amine  [mL] | Oleic acid  [mL] | Injection temperature [°C] |
| --- | --- | --- | --- |
| Oleylamine | 0.500 (1.5 mmol) | 0.050 (0.15 mmol) | 160 |
| Dodecylamine | 0.350 (1.5 mmol) | 0.150 (0.45 mmol) | 170 |
| Decylamine | 0.303 (1.5 mmol) | 0.100 (0.30 mmol) | 170 |
| Octylamine | 0.250 (1.5 mmol) | 0.150 (0.45 mmol) | 170 |
| Hexylamine | 0.200 (1.5 mmol) | 0.075 (0.23 mmol) | 175 |

# 2) Rocking curve analysis method

Rocking curve measurements were performed to study the angular misalignment of the nanocrystals.
For our analysis, we neglected any contribution that could be related to size dispersion or strain. The former was not considered in our analysis primarily due to its static effect: even if some of the room-temperature broadening might be attributed to inhomogeneous particle size, such contribution would remain unchanged throughout the temperature ramp and therefore would not affect our observation of the phase transition. Besides, we believe that particle size dispersion is low in our samples on the empirical basis of:

- High quality superlattices like the ones we studied in this work require very uniform nanocrystals to grow properly;
- The size-selection process demonstrated in Figure 4 of the Main Text further narrows the particle size distribution at the center of the substrate, that is where the XRD signal was collected.

For what concerns the contribution of strain, the traditional estimation via the Williamson-Hall analysis cannot be applied in the case of a highly oriented sample like the superlattices studied here. In fact, only two peaks can be observed and their profile is heavily influenced by multilayer interference, which would make any evaluation of their intrinsic broadening unreliable. Therefore, we opted for a different approach.


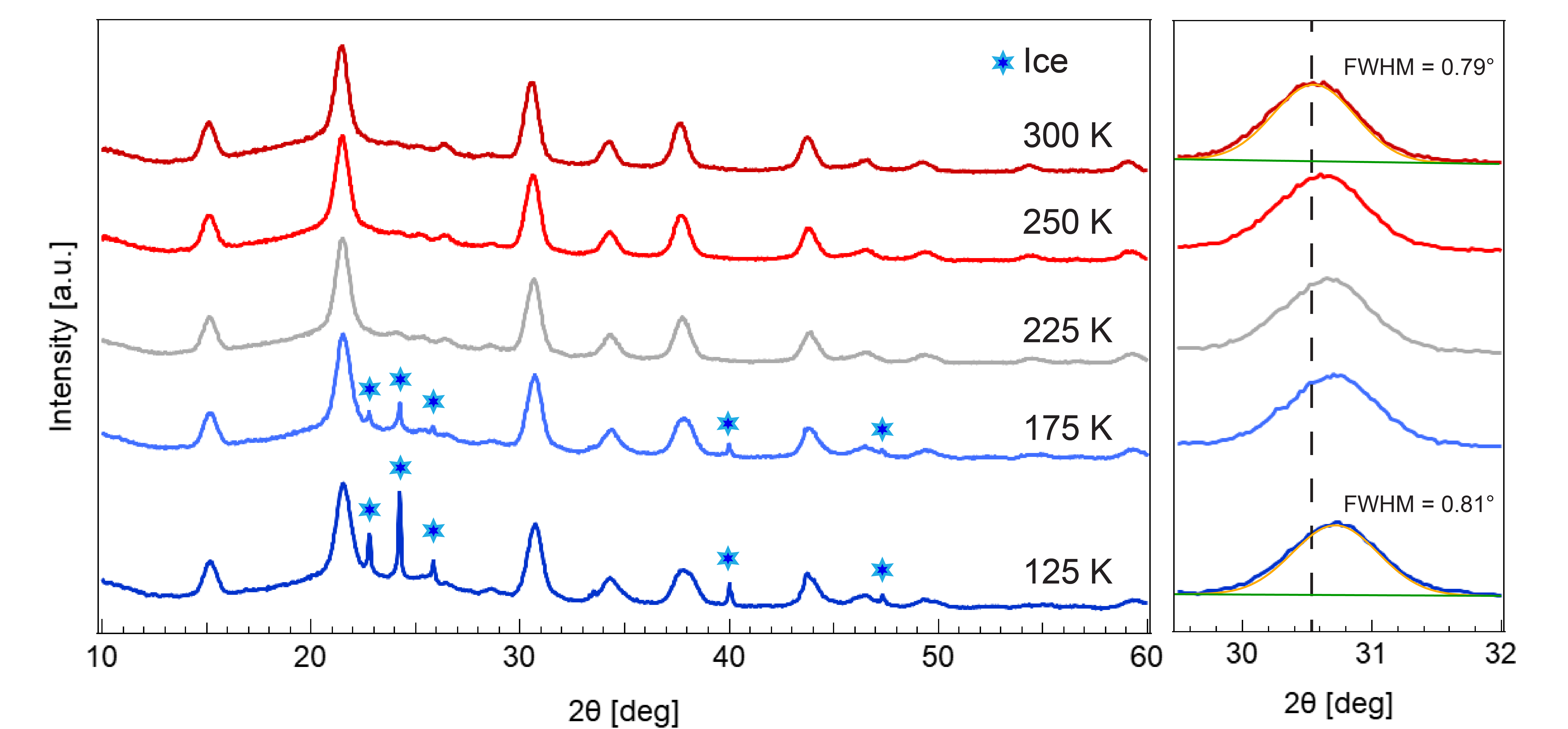


**Figure S1**. θ:2θ X-ray diffraction patterns of a film of randomly oriented CsPbBr_3_ nanocrystals obtained by mixing the nanocrystals with amorphous silica. Bragg peaks shift towards higher angle with decreasing temperature due to the expected thermal contraction of CsPbBr_3_ crystal structure parameter “d”.

First, we note that non-assembled films of CsPbBr_3_ nanocrystals show just a minor Bragg peak broadening upon cooling to cryogenic temperatures (e.g. +3% for the (200) pseudocubic Bragg peak of CsPbBr_3_ nanocrystals absorbed on silica powders to prevent self-assembly, Figure S1). This can be largely attributed to the known tendency of CsPbBr_3_ to become more anisotropic upon cooling, resulting in a wider orthorhombic split of the Bragg reflections that contribute to the pseudocubic (200) peak (see^[1]^ for reference lattice parameters of bulk CsPbBr_3_ at different temperatures). Therefore, we must assume that any inhomogeneous strain contribution to the rocking curves is exclusive to superlattices, which would point to a ligand-induced strain. However, the rocking curve of C_18_-sample is significantly broader than that of C_8_-sample already at room temperature, where both ligands are still liquid and have not stiffened yet, which points against strain being a significant factor in this comparison.

For data acquisition, the X-ray source and detector were positioned at a 2θ angle corresponding to the maximum of the (200) Bragg peak of CsPbBr_3_ (for example 2θ = 30.50° at 300 K), and were rocked simultaneously up to 12.9° in both clockwise and anticlockwise directions while keeping 2θ constant. For data analysis, the rocking curves were fitted with a simulated curve profile *S.R. (ω)*, obtained from the convolution of a Gaussian profile *G* (*ω_0_, σ_ω_*) with the calculated rocking curve of an individual nanocrystal *R_NC_* (*L, ω, 2*θ):

$S.R.(\omega)=G\left( \omega_{0}, \sigma_{\omega} \right)*R_{NC}(L,\omega,2\theta)$ Eq. (I)

Here, *G* (*ω_0_, σ_ω_*) describes the tilting of nanocrystals away from the normal vector to the substrate surface. It is centered around ω_0_ = 0°, which corresponds to nanocrystals lying perfectly flat on the substrate, and its standard deviation *σ_ω_* measures the angular disorder in superlattices.

Instead, *R_NC_* (*L, ω,* θ) represents the intensity diffracted at a given 2θ angle by an individual nanocrystal as a function of its tilt angle *ω* with respect to the ideal orientation (*ω = 0*). This has been calculated theoretically by building an atomistic model of a cubic-shaped CsPbBr_3_ nanocrystal with edge length *L*, and then computing the diffracted intensity at a given scattering vector modulus ($q=4\pi sin\left( \theta\right)/\lambda_{XRD}$) through the following equation:

$I=\left| \sum_{j} f_{g}e^{-iq\cdot z_{j}} \right|^{2}$ Eq. (II)

where the summation iterates over all atoms in the nanocrystal model, *f_j_* is the atomic form factor of the *j^th^* atom, *z_j_* is its vertical coordinate in the model, and *q* is the scattering vector.

As an example, Figure S2a shows a 6.5 nm nanocrystal model tilted along the *x*-axis (here seen in its *yz*-projection), while Figure S2b shows the corresponding *R_NC_* profiles calculated for q = 1.09 Å^-1^ (first CsPbBr_3_ Bragg peak) and q = 2.17 Å^-1^ (second CsPbBr_3_ Bragg peak). Notably, the intensity drop is faster as the order of Bragg reflections increases. Finally, the calculated *R_NC_* profile is convoluted with *G* to produce *S.R.* To extract the tilting disorder parameter *σ_ω_*, the program simply optimizes *G* (*ω_0_, σ_ω_*) to match the experimental data through a least-square fitting procedure.


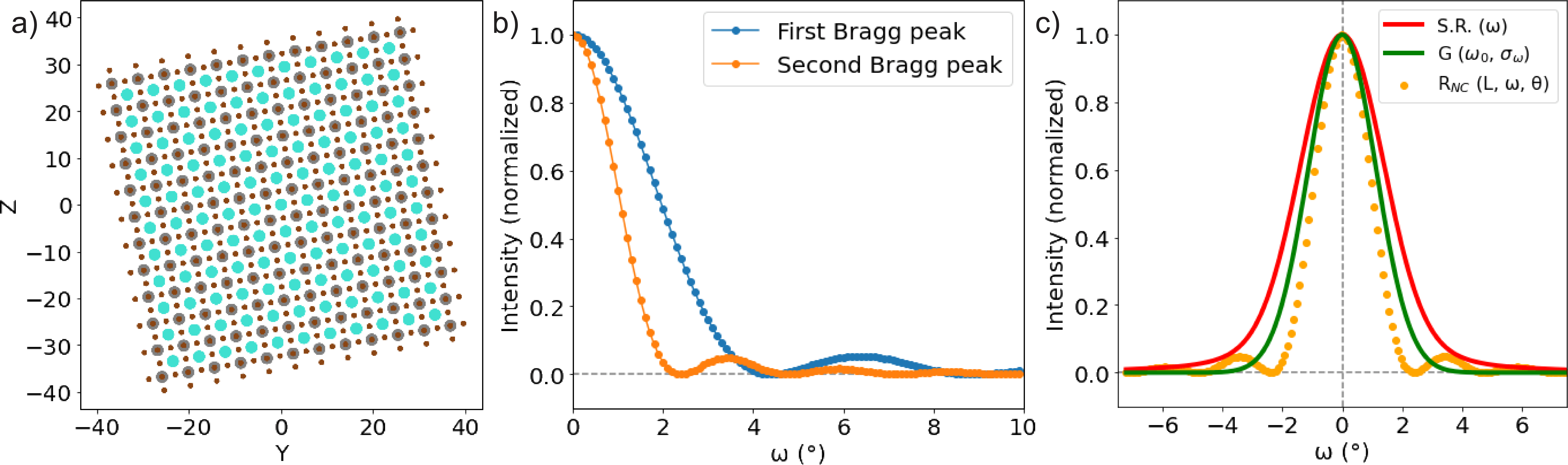


**Figure S2**. **Rocking curve analysis example.** a) Atomistic model of a 6.5 nm CsPbBr_3_ nanocrystal (red = Br, cyan = Cs, grey = Pb), tilted at an angle of *ω =* 9.9° around the *x*-axis of the laboratory reference system. b) Calculated rocking curve profiles for the same nanocrystal model shown in Panel (a), for its first (q = 1.09 Å^-1^, blue) and second (q = 2.17 Å^-1^, orange) Bragg peaks. c) Convolution of the Gaussian tilting profile *G* with the second-peak rocking curve *R_Nc_* shown in panel (b) to produce a simulated rocking curve *S.R*.

# 3) XRD on oleylamine-capped nanocrystal superlattices

Table S2 and Figure S3 summarize the results of the multilayer diffraction fit on the XRD patterns of oleylamine-capped nanocrystal superlattices, performed following the procedures detailed in Refs.^[2–4]^ Table S3 and Figure S4 summarize the results of the rocking curve fit for the same samples and temperatures, performed as described in paragraph 2.

**Table S2. Multilayer diffraction fits for oleylamine-capped nanocrystal superlattices.** Structure parameters extracted via the multilayer diffraction fit of temperature-dependent XRD patterns of superlattices grown from CsPbBr_3_ nanocrystals capped with oleylamine and oleic acid (see Figure S3). Parameters: d = nanocrystal lattice constant; L = interparticle distance (surface to surface); σ_L_ = stacking disorder parameters (see Refs. ^[1–3]^) ; N = nanocrystal thickness; σ_N_ = nanocrystal thickness distribution.

| **Temperature [K]** | **d**  **[Å]** | **L**  **[Å]** | **σ_L_**  **[Å]** | **N**  **[planes]** | **σ_N_**  **[planes]** |
| --- | --- | --- | --- | --- | --- |
| **Cooling** | | | | | |
| **300 K** | 5.843 ±  0.002 | 36.903 ±  0.051 | 1.280 ±  0.016 | 12.880 ±  0.187 | 2.459 ±  0.298 |
| **250 K** | 5.834 ±  0.002 | 38.105 ±  0.041 | 1.888 ±  0.015 | 12.617 ±  0.143 | 2.862 ±  0.250 |
| **225 K** | 5.836 ±  0.003 | 35.740 ±  0.064 | 1.577 ±  0.019 | 12.601 ±  0.004 | 2.208 ±  0.217 |
| **175 K** | 5.820 ± 0.  003 | 34.201 ±  0.108 | 1.881 ±  0.023 | 13.246 ±  0.183 | 1.513 ±  0.367 |
| **125 K** | 5.806 ±  0.003 | 33.851 ±  0.093 | 1.898 ±  0.021 | 13.269 ±  0.205 | 1.818 ±  0.376 |
| **90 K** | 5.801 ±  0.003 | 33.943 ±  0.116 | 1.885 ±  0.018 | 12.987 ±  0.223 | 1.921 ±  0.374 |
| **Re-heating** | | | | | |
| **125 K** | 5.812 ±  0.003 | 34.075 ±  0.116 | 1.856 ±  0.021 | 12.964 ±  0.206 | 2.155 ±  0.375 |
| **175 K** | 5.824 ±  0.003 | 34.397 ±  0.117 | 1.874 ±  0.021 | 13.029 ±  0.183 | 1.557 ±  0.345 |
| **225 K** | 5.834 ±  0.002 | 35.632 ±  0.067 | 1.618 ±  0.019 | 12.601 ±  0.006 | 2.264 ±  0.211 |
| **250 K** | 5.835 ±  0.002 | 37.704 ±  0.042 | 1.869 ±  0.016 | 12.602 ±  0.009 | 3.059 ±  0.158 |
| **300 K** | 5.847 ±  0.003 | 37.099 ±  0.063 | 1.279 ±  0.014 | 13.006 ±  0.162 | 2.322 ±  0.284 |


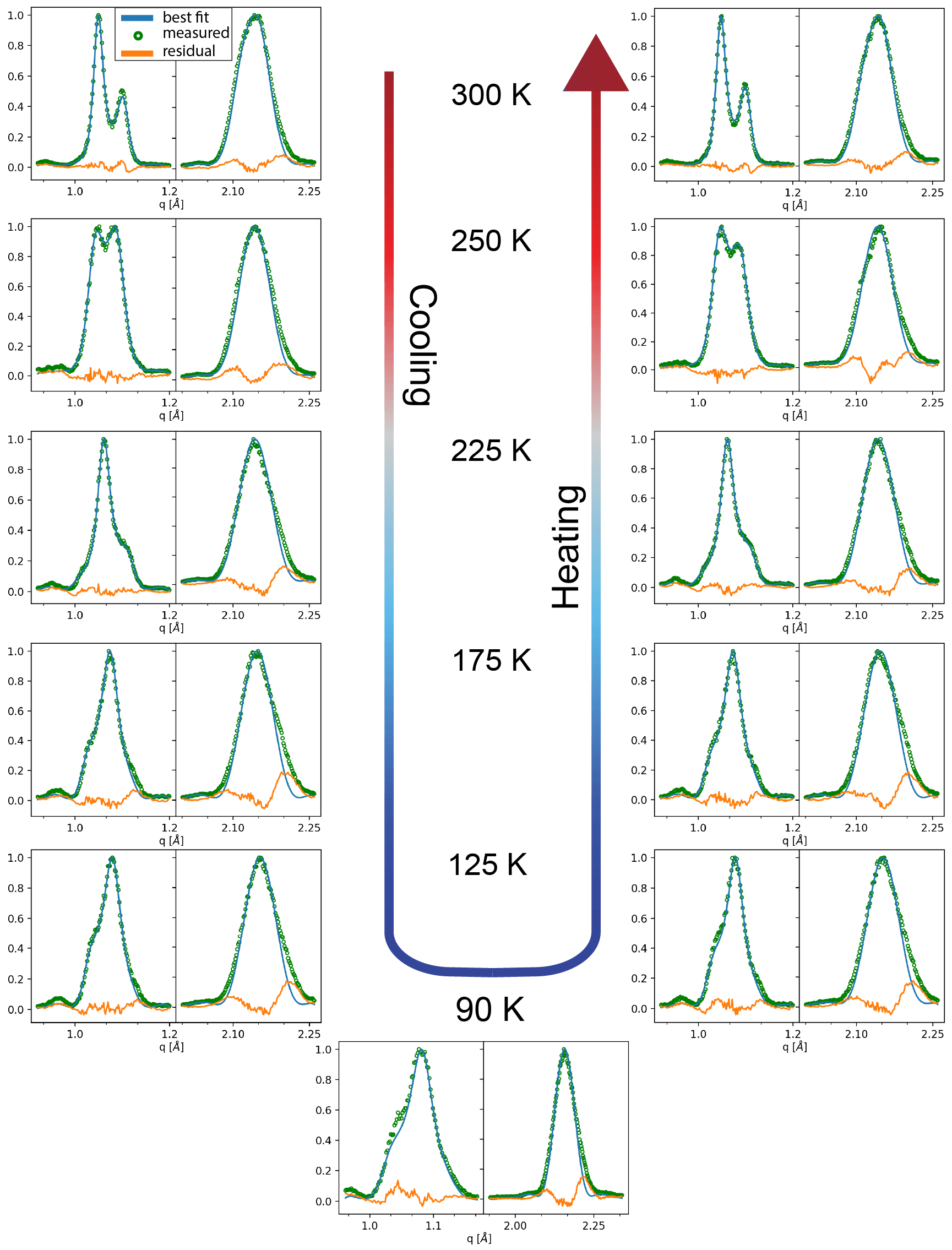


**Figure S3. Multilayer diffraction fits for oleylamine-capped nanocrystal superlattices*.*** Temperature-dependent evolution of the first and second Bragg peak profiles for superlattices grown from CsPbBr_3_ nanocrystals capped with oleylamine and oleic acid. Experimental data is shown in green, the corresponding multilayer diffraction fit is shown in blue and the residual curve is plotted in orange. The temperature series has been collected starting from the top-left pattern and proceeding counter-clockwise, first with cooling (left) and then with re-heating back to room temperature (right).

**Table S3. Rocking curve fits for oleylamine-capped nanocrystal superlattices.** Nanocrystal tilting disorder parameters as extracted by fitting the temperature-dependent rocking curves of superlattices grown from CsPbBr_3_ nanocrystals capped with oleylamine and oleic acid (see Figure S4). σ_L_ = nanocrystal tilting disorder.

| **Temperature**  **[K]** | **σ_ω_**  **[° θ]** | **Temperature**  **[K]** | **σ_ω_**  **[°θ]** |
| --- | --- | --- | --- |
| **Cooling** | | **Re-heating** | |
| **300 K** | 3.686 ± 0.066 | **300 K** | 3.686 ± 0.006 |
| **250 K** | 3.971 ± 0.024 | **250 K** | 3.926 ± 0.024 |
| **225 K** | 4.269 ± 0.027 | **225 K** | 4.286 ± 0.078 |
| **175 K** | 4.394 ± 0.013 | **175 K** | 4.421 ± 0.014 |
| **125 K** | 4.384 ± 0.019 | **125 K** | 4.369 ± 0.015 |
| **90 K** | 4.383 ± 0.001 |  |  |

**Figure S4. Rocking curve fits for oleylamine-capped nanocrystal superlattices.** Top-row patterns have been collected while cooling the sample down to 90 K, while bottom-row patterns have been collected while heating the sample from 90 K to 300 K.


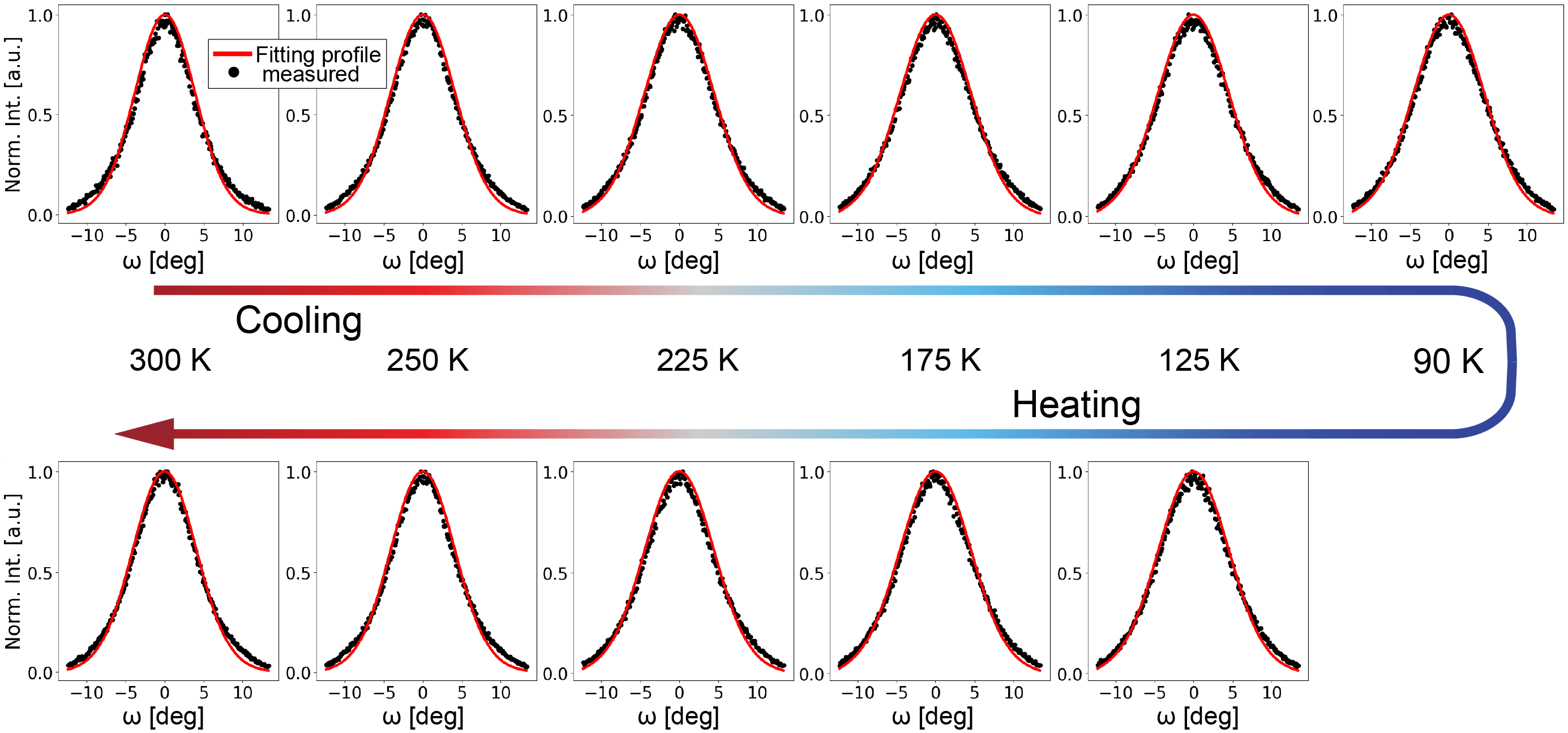


# 4) XRD on octylamine-capped nanocrystal superlattices

Table S4 and Figure S6 summarize the results of the multilayer diffraction fit on the XRD patterns of octylamine-capped nanocrystal superlattices, performed following the procedures detailed in Refs. ^[1–3]^. Table S5 and Figure S7 summarize the results of the rocking curve fit for the same samples and temperatures, performed as described in paragraph 2.

Note that during the cooling process, slightly different structural dynamics are visible for different fractions of the sample (either different superlattices or different domains within the same superlattice). Indeed, at T = 250 K and T = 225 K we observed an additional peak within the fine structure of first Bragg reflection, which we interpret as partially disordered domains that temporarily lost their structural coherence. Based on the area of those signals, we estimate this phenomenon to involve ∼20/30% of the nanocrystals in the sample (Figure S5). However, such behavior is transient, as the XRD pattern measured at 175 K can be again fitted with the sole contribution of multilayer diffraction from highly ordered superlattices. Moreover, the phenomenon is fully reversible, and it occurs again when the sample is heated back to room temperature. We hence speculate that this transient loss of structural coherence is part of the phase transition mechanism of the sample, and not just a cooling-induced amorphization.


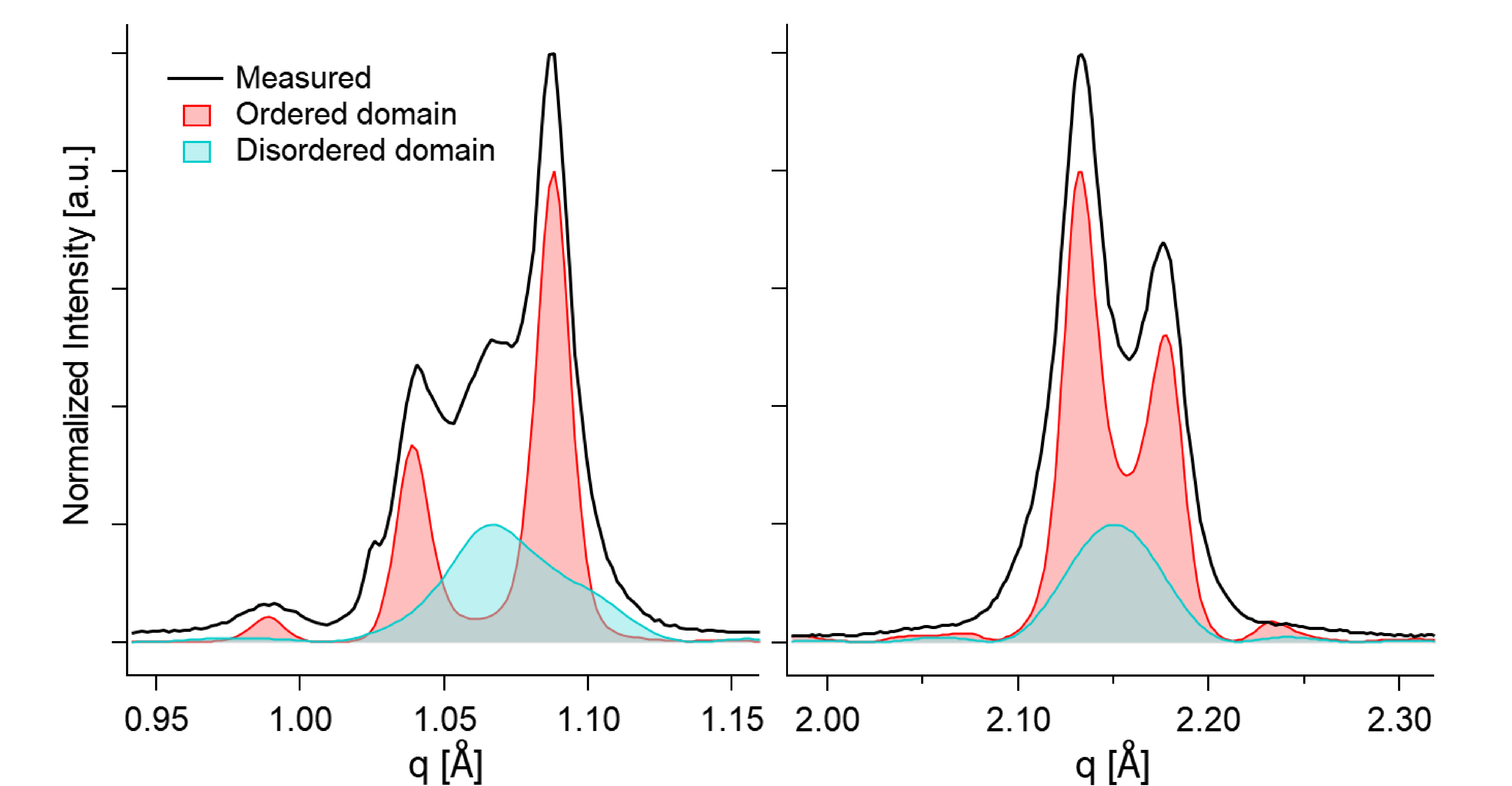


**Figure S5. Example of a multilayer fitting performed accounting for two contributions.** XRD pattern of octylamine-capped nanocrystal superlattices measured at 250 K while heating. Two contributions are required to fit the pattern, one ordered (σ_L_ = 0.65 Å, ≈ 80 %, red traces) and one disordered (σ_L_ = 1.96 Å, ≈ 20 %, light blue traces).

**Table S4. Multilayer diffraction fits for octylamine-capped nanocrystal superlattices.** Structure parameters extracted via the multilayer diffraction fit of temperature-dependent XRD patterns of superlattices grown from CsPbBr_3_ nanocrystals capped with octylamine and oleic acid (see Figure S6). Parameters: d = nanocrystal lattice constant ; L = interparticle distance (surface to surface) ; σ_L_ = stacking disorder parameters (see Refs. ^[1–3]^) ; N = nanocrystal thickness ; σ_N_ = nanocrystal thickness distribution. Rows marked in grey indicate the co-presence of a low-order contribution, whose estimate volume percentage is shown in parenthesis under the temperature.

| **Temperature [K]** | **d**  **[Å]** | **L**  **[Å]** | **σ_L_**  **[Å]** | **N**  **[planes]** | **σ_N_**  **[planes]** |
| --- | --- | --- | --- | --- | --- |
| **Cooling** | | | | | |
| **300 K** | 5.855 ± 0.001 | 33.177 ±  0.017 | 0.628 ±  0.008 | 18.011 ±  0.155 | 1.081 ±  0.057 |
| **250 K**  **(23% disordered)** | 5.841 ± 0.009 | 33.113 ±  0.026 | 0.605 ±  0.014 | 18.085 ±  0.221 | 1.062 ±  0.250 |
| **225 K**  **(27% disordered)** | 5.821 ± 0.008 | 33.593 ±  0.017 | 0.7581 ±  0.015 | 18.206 ±  0.146 | 1.208 ±  0.217 |
| **175 K** | 5.816 ± 0.008 | 27.198 ±  0.041 | 1.492 ±  0.020 | 18.000 ±  0.001 | 4.898 ±  0.339 |
| **125 K** | 5.796 ± 0.005 | 26.936 ±  0.046 | 1.489 ±  0.0042 | 17.800 ±  0.002 | 5.114 ±  0.249 |
| **Re-heating** | | | | | |
| **175 K** | 5.819 ± 0.003 | 27.337 ±  0.044 | 1.469 ±  0.027 | 17.900 ±  0.000 | 5.305 ±  0.376 |
| **225 K**  **(27% disordered)** | 5.822 ± 0.002 | 33.184 ±  0.051 (73 %) | 0.723 ±  0.039 (73 %) | 18.063 ±  0.112 | 2.328 ±  0.210 |
| **250 K**  **(20% disordered)** | 5.828 ± 0.002 | 33.253 ±  0.019 (80 %) | 0.650 ±  0.019 (80 %) | 17.988 ±  0.250 | 1.068 ±  0.152 |
| **300 K** | 5.851 ± 0.003 | 33.223 ±  0.017 | 0.696 ±  0.008 | 17.823 ±  0.023 | 1.022 ±  0.164 |

**
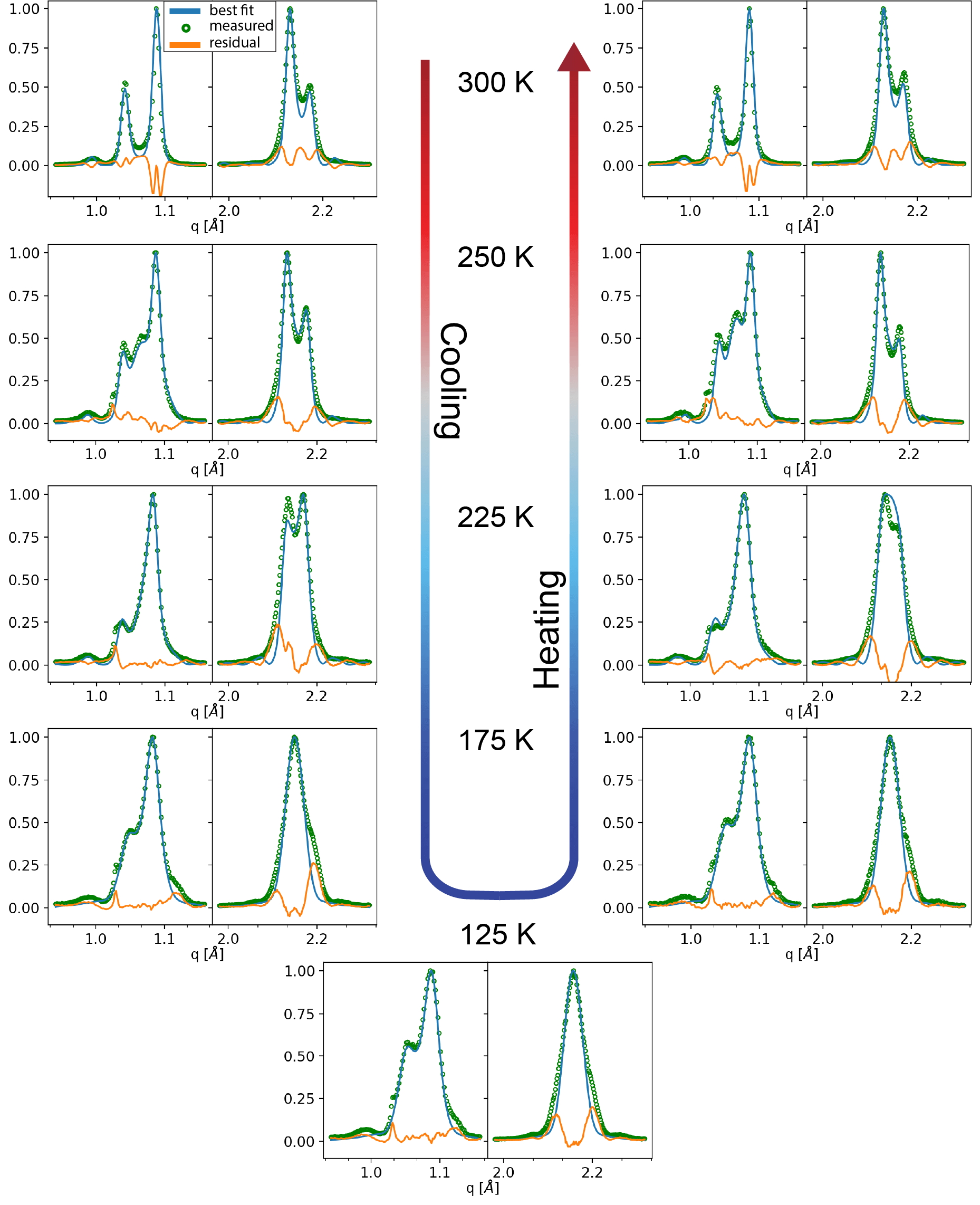
**

**Figure S6. Multilayer diffraction fits for octylamine-capped nanocrystal superlattices*.*** Temperature-dependent evolution of the first and second Bragg peak profiles for superlattices grown from CsPbBr_3_ nanocrystals capped with octylamine and oleic acid. Experimental data is shown in green, the corresponding multilayer diffraction fit is shown in blue and the residual curve is plotted in orange. The temperature series has been collected starting from the top-left pattern and proceeding counter-clockwise, first with cooling (left) and then with re-heating back to room temperature (right).

**Table S5. Rocking curve fits for octylamine-capped nanocrystal superlattices.** Nanocrystal tilting disorder parameters as extracted by fitting the temperature-dependent rocking curves of superlattices grown from CsPbBr_3_ nanocrystals capped with octylamine and oleic acid (see Figure S7). σ_L_ = nanocrystal tilting disorder.

| **Temperature**  **[K]** | **σ_ω_**  **[° θ]** | **Temperature**  **[K]** | **σ_ω_**  **[°θ]** |
| --- | --- | --- | --- |
| **Cooling** | | **Re-heating** | |
| **300 K** | 1.646 ± 0.000 | **300 K** | 2.904 ± 0.037 |
| **250 K** | 2.780 ± 0.064 | **250 K** | 3.046 ± 0.108 |
| **225 K** | 3.234 ± 0.000 | **225 K** | 3.194 ± 0.028 |
| **175 K** | 3.361 ± 0.070 | **175 K** | 3.336 ± 0.047 |
| **125 K** | 3.410 ± 0.021 |  |  |

**Figure S7. Rocking curve fits for octlyamine-capped nanocrystal superlattices.** Top-row patterns have been collected while cooling the sample down to 90 K, while bottom-row patterns have been collected while heating the sample from 90 K to 300 K.


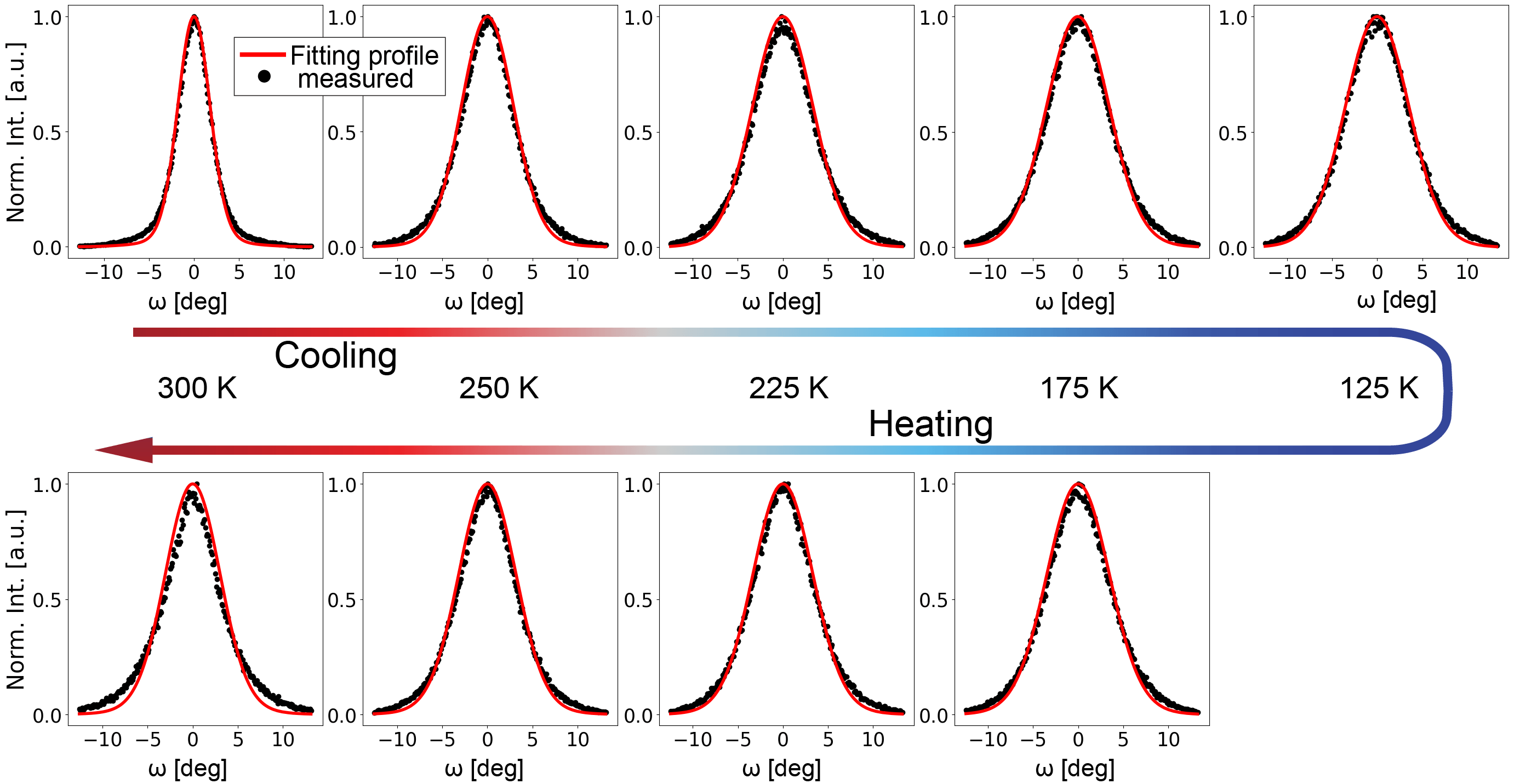


# 5) Fitting results of nanocrystals passivated with different amines and interparticle spacing calculation

**Table S6.** Multilayer diffraction and rocking curves fitting results for nanocrystal superlattice passivated with a combination of oleic acid and amines of different lengths.

|  | **Multilayer diffraction** | | | | | **Rocking Curves** |
| --- | --- | --- | --- | --- | --- | --- |
| **Passivation: Oleic Acid +** | **d**  **[Å]** | **L**  **[Å]** | **σ_L_**  **[Å]** | **N**  **[planes]** | **σ_N_**  **[planes]** | **σ_ω_**  **[°θ]** |
| **Oleylamine** | 5.854 ± 0.008 | 39.560 ± 0.056 | 1.293 ± 0.000 | 13.619 ± 0.009 | 0.475 ± 0.267 | 3.645 ± 0.066 |
| **Dodecylamine** | 5.866 ± 0.004 | 38.270 ± 0.031 | 1.122 ± 0.015 | 14.371 ± 0.077 | 0.245 ± 0.112 | 3.550 ± 0.066 |
| **Decylamine** | 5.861 ± 0.001 | 33.929 ± 0.015 | 0.803 ± 0.006 | 16.823 ± 0.161 | 0.753 ± 0.394 | 2.292 ± 0.101 |
| **Octylamine** | 5.855 ± 0.001 | 33.463 ±  0.015 | 0.617 ±  0.008 | 18.011 ±  0.155 | 1.081 ±  0.057 | 1.563 ± 0.070 |
| **Hexylamine** | 5.854 ± 0.002 | 28.752 ± 0.030 | 0.426 ± 0.001 | 22.695 ± 4.313 | 3.808 ± 2.258 | 1.556 ± 0.101 |

**Interparticle spacing calculation.** Following previously reported calculations for the length of organic ligands,^[5–7]^ the maximum end-to-end length for saturated hydrocarbon chains comprising *n* carbon atoms is:

$h_{max} = nr_{0}\cos\left( \frac{\alpha}{2} \right)+r_{1}=0.128\cdot n+0.2 [nm]$ Eq. (III)

where $r_{0}$ = 0.154 nm is the C-C bond distance, $\alpha=180^{\circ}-112^{\circ}=68^{\circ}$ is the C – C – C angle and $r_{1}=0.2$ nm is the radius of the end methyl group. Here, the head amine group is counted as an extra C – C bond. The maximum end-to-end length for a hydrocarbon chain consisting of (*n-1*) single bonds plus one unsaturated bond is:

$h_{max} \approx\left[ \left( n-1 \right)r_{0}\cos\left( \frac{\alpha}{2} \right)+r_{1} \right]\cos\left( \omega\right)+r_{dd}$ Eq. (IV)

where $r_{dd}$ = 0.133 nm is the double bond length and $\omega=\frac{\pi}{3}-\frac{\alpha}{2}=24.5$°. The ligand-nanocrystal bond length $l_{k}$ = 0.255 nm, is accounted here as a part of the ligand length. By combining the maximum length of different ligand molecules, we can calculate the maximum interparticle separation $L_{max}$ as:

$L_{max}= h_{max}^{ligand 1}+h_{max}^{ligand 2}+l_{k}*2$ Eq. (V)

$\xi=\frac{\sigma}{\sigma_{Max}}$ Eq. (VI)

$\xi^{'}=\frac{L}{L_{max}}$ Eq. (VII)

where $\xi$ is the dimensionless ligand grafting density on the surface of a nanocrystal, $\sigma$is the experimental grafting density and $\sigma_{Max}$ is the maximum theoretical grafting density.^[4–6]^

In Table S7, we report calculations performed by assuming that the nanocrystals are passivated exclusively by one type of surfactant, that is the amine, and that both nanocrystals contribute with the same ligand type to Equation IV. We also assume that a grafting density $\xi$ = 1, corresponding to a fully passivated nanocrystal surface, would result in the ligands being fully extended and therefore produce the maximum interparticle distance possible with that passivation, that is $L_{max}$.

By comparing such number with the interparticle distance as extracted from multilayer diffraction, we demonstrate that a passivation composed exclusively by amines is not corresponding to the experimental reality, at least for amines shorter than *n = 11* (that is, decylamine), as the measured interparticle distance would require a grafting density *ξ > 1*. We thus conclude that oleic acid is also present on the surface of nanocrystals, which we also confirmed by NMR experiments (see paragraph 6).

**Table S7. Experimental vs calculated interparticle spacing.** Interparticle separation distance as measured via multilayer diffraction (*L_XRD_*), compared with estimates obtained by considering the ligands length chain (*L*_max_). The dimensionless grafting density *ξ* indicates the fraction of surface coverage that would be required to make the measured and estimated interparticle distances converge. Note that *ξ > 1* is physically unattainable, indicating that the proposed passivation model is incompatible with7 experimental reality.

| Passivation | $\boldsymbol{L}_{\boldsymbol{max}}$ [nm] | $\boldsymbol{L}_{\boldsymbol{XRD}}$ [nm] | Grafting density *ξ* |
| --- | --- | --- | --- |
| Oleylamine (*n* = 19) | 5.33 | 3.96 | 0.74 |
| Dodecylamine (*n* = 13) | 4.03 | 3.82 | 0.94 |
| Decylamine (*n* = 11) | 3.62 | 3.39 | 0.94 |
| Octylamine (*n* = 9) | 3.07 | 3.35 | 1.09 |
| Hexylamine (*n* = 7) | 2.59 | 2.87 | 1.11 |

# 6) Nuclear Magnetic Resonance (NMR) analysis

All NMR experiments were performed at 298 K on a Bruker (Bruker, Rheinstetten, Germany) Avance III spectrometer, fitted with a 5 mm QCI cryoprobe. The nanocrystals were synthesized and purified following the procedure described in the experimental section in the main text. After purification, the nanocrystals were dried under nitrogen flow for ≈ 1 hour, and then suspended in 550 µL of toluene-d_8_, transferred into 5mm tubes, for measuring the NMR signal of ligands while attached to the nanocrystals surface. Once completed, the same nanocrystals were recovered from toluene-d_8_ by drying under a nitrogen flow, and then nanocrystals were dissolved in 200 µL of DMSO-d_6_  (DiMethyl SulfOxide) and transferred into 3 mm disposable SampleJet (Bruker) tubes to perform measurements on the now free ligands (the nanocrystal core is soluble in DMSO).

Before each NMR acquisition, automatic routines for matching, tuning and optimization of the resolution were performed. The 90°was automatically calculated on each sample tube.^[8]^ For ^1^H NMR spectra (both in toluene-d_8_ and in DMSO-d_6_ ), 128 transients were accumulated without steady scans, at a fixed receiver gain (9), with 65536 complex points and an inter-pulses delay of 30 s, over a spectral width of 20.83 ppm centered at 6.18 ppm. An exponential smoothing function equivalent to 0.3 Hz was applied to FIDs before Fourier transformed. The signal integration was performed using Mnova software. ^1^H-^1^H NOESY ([Nuclear Overhauser Effect Spectroscopy) experiment (*noesygpphpp*, Bruker’s library) was acquired with 64 scans, after 16 dunny ones, 2048 digits points, 256 increments, using 300 ms of mixing time, over a spectral width of 13.02 ppm (offset positioned at 6.18 ppm).](https://www.sciencedirect.com/topics/materials-science/nuclear-overhauser-effect-spectroscopy) ^1^H-^1^H COSY (COrrelated SpectroscopY) spectrum (*cosygpmqf*, Bruker’s library) was run with 16 scans, 16 steady ones, 2048 digits points and 128 increments, over a spectral width of 13.02 ppm, centered at 6.18 ppm.

^1^H–^13^C HSQC [(Heteronuclear Single-Quantum Coherence Spectroscopy) experiment (*hsqcetgppsi2*, Bruker’s library) was performed by accumulating 8 transients, after 16 steady scans, with 1024 digits, 256 increments and a ^1^J_CH_ of 145 Hz, over a spectral width of 13 and 165 ppm for ^1^H and ^13^C respectively, with the offset at 6.69 and 75 ppm.](https://www.acronymfinder.com/Heteronuclear-Single_Quantum-Coherence-Spectroscopy-(HSQC).html)^1^H–^13^C HMBC [(Heteronuclear Multiple Bond Correlation) experiment (*hmbcgplpndqf*, Bruker’s library) was carried out with 128 transients, 16 dunny scans, 4096 digit points, 128 increments and a ^1^ J_CH_ long range of 10 Hz, over a spectra width of 15.15 and 220 ppm for ^1^H and ^13^C, respectively, with the offset at 7 and 100 ppm.](https://www.acronymfinder.com/Heteronuclear-Single_Quantum-Coherence-Spectroscopy-(HSQC).html) All spectra were referred to not deuterated residual solvent peaks of toluene-d_7_, calibrated at 7.09 ppm, and DMSO-d_5_ set at 2.50 and 39.5 ppm for ^1^H and ^13^C, respectively.

**Table S8. Oleic acid/amine ratio quantified by NMR.** The quantification of ligands present in the solutions for superlattice assembly were performed via the analysis of quantitative ^1^H-NMR spectra (see Figures S9a and S11a), collected after dissolving the nanocrystals in DMSO-d_6_.

| **Nanocrystals capping** | **Reference signal**  **(assigned ligand)** | **Peak integral** | **Molar ratio** |
| --- | --- | --- | --- |
| **Octylamine + Oleic acid** | -**H**C=C**H**- (oleic acid) | 200 | 0.38 |
|  | C**H_2_** – N (octylamine) | 325 | 0.62 |
| **Oleylamine + Oleic acid** | -**H**C=C**H**-  (oleic acid + oleylamine) | 200 | 0.11 (oleic acid)  0.89 (oleylamine) |
|  | C**H_2_** – N (oleylamine) | 178 | 0.89 |

**
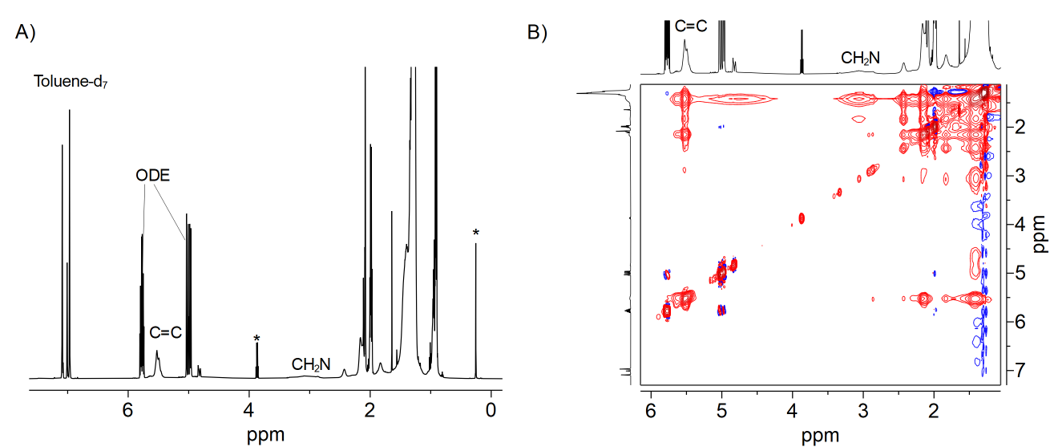
**

**Figure S8. NMR analysis on oleylamine-capped nanocrystals in toluene-d_8_.** a) ^1^H NMR and b) ^1^H-^1^H 2D NOESY NMR spectra of nanocrystals prepared by using a mixture of oleylamine and oleic acid as surfactants. Diagnostic signals for the presence of oleic acid and oleylamine are the vinylic HC=CH signal at 5.6 ppm and the CH_2_N at 3.0 ppm, respectively. The broadening of both diagnostic signals is a signature of surfactants interacting with the nanocrystals surface. This is further confirmed by the negative (red) NOE cross-peaks in the NOESY experiment, characteristic of species with slow tumbling regime in solution due to the binding with the nanocrystals.

**
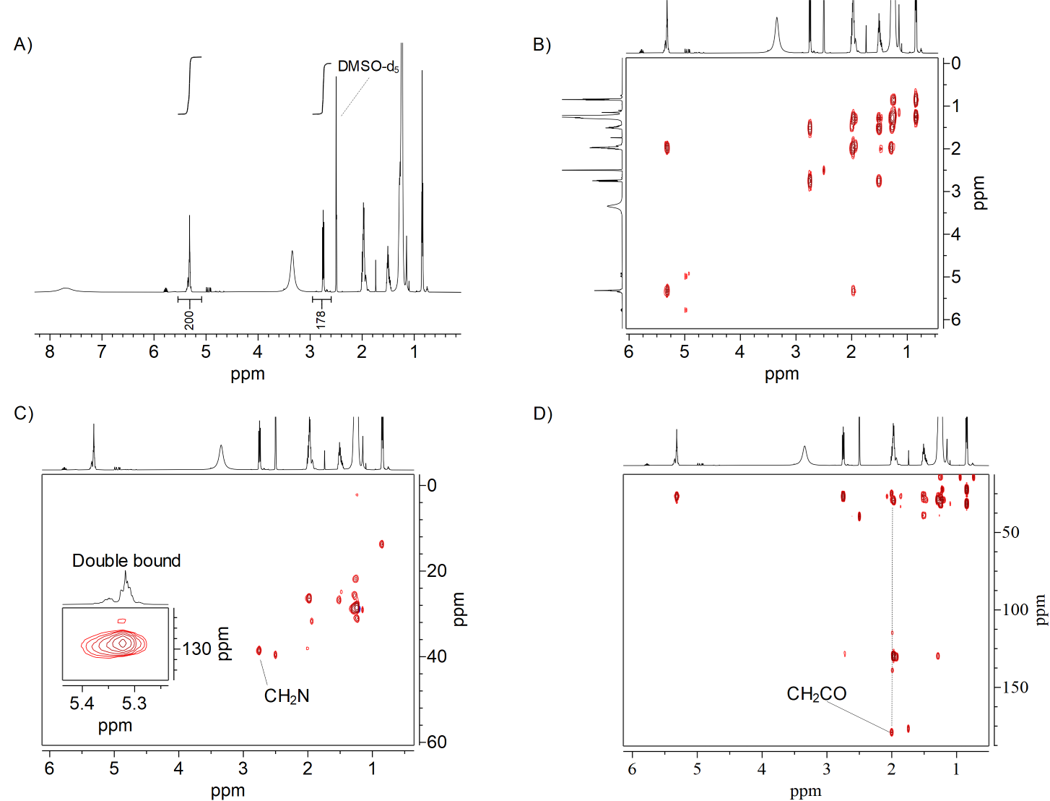
**

**Figure S9. NMR analysis on oleylamine-capped nanocrystals in DMSO-d_6_.** a) Quantitative ^1^H NMR performed on a solution of free ligands in DMSO-d6, after the CsPbBr_3_ inorganic core of nanocrystals has been dissolved. The attribution of ligand-diagnostic peaks (HC=CH signal at 5.32 ppm for oleic acid and CH_2_N at 3.0 ppm for oleylamine) was confirmed via ^1^H-^1^H COSY (b), ^1^H-^13^C HSQC (d), and ^1^H-^13^C HMBC spectroscopies. The relative concentration of ligands was determined by integral ratio between the CH_2_N triplet of oleylamine at 2.7 ppm and the HC=CH signal of oleic acid, obtained by subtracting the oleylamine contribution to the signals in the double region at ∼5.3 ppm, which can be attributed to both the oleylamine and the oleic acid due to the presence of a double bond in their aliphatic chain.


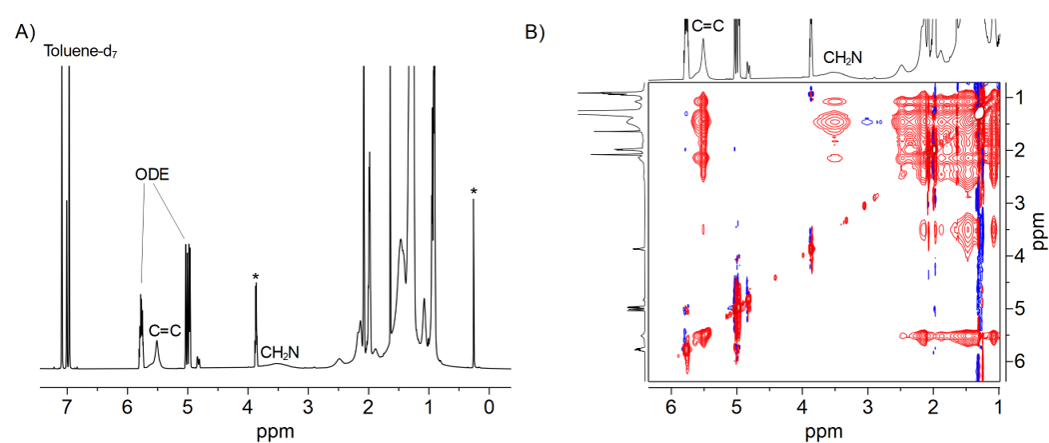


**Figure S10. NMR analysis on octylamine-capped nanocrystals in toluene-d_8_.** a) ^1^H NMR and b) ^1^H-^1^H 2D NOESY NMR spectra of nanocrystals prepared by using a mixture of oleylamine and oleic acid as surfactants. Diagnostic signals for the presence of oleic acid and octylamine are the vinylic HC=CH signal at 5.6 ppm and the CH_2_N at 3.5 ppm, respectively. The broadening of both diagnostic signals is a signature of surfactants interacting with the nanocrystals surface. This is further confirmed by the negative (red) NOE cross-peaks in the NOESY experiment, characteristic of species with slow tumbling regime in solution due to the binding with the nanocrystals.

**
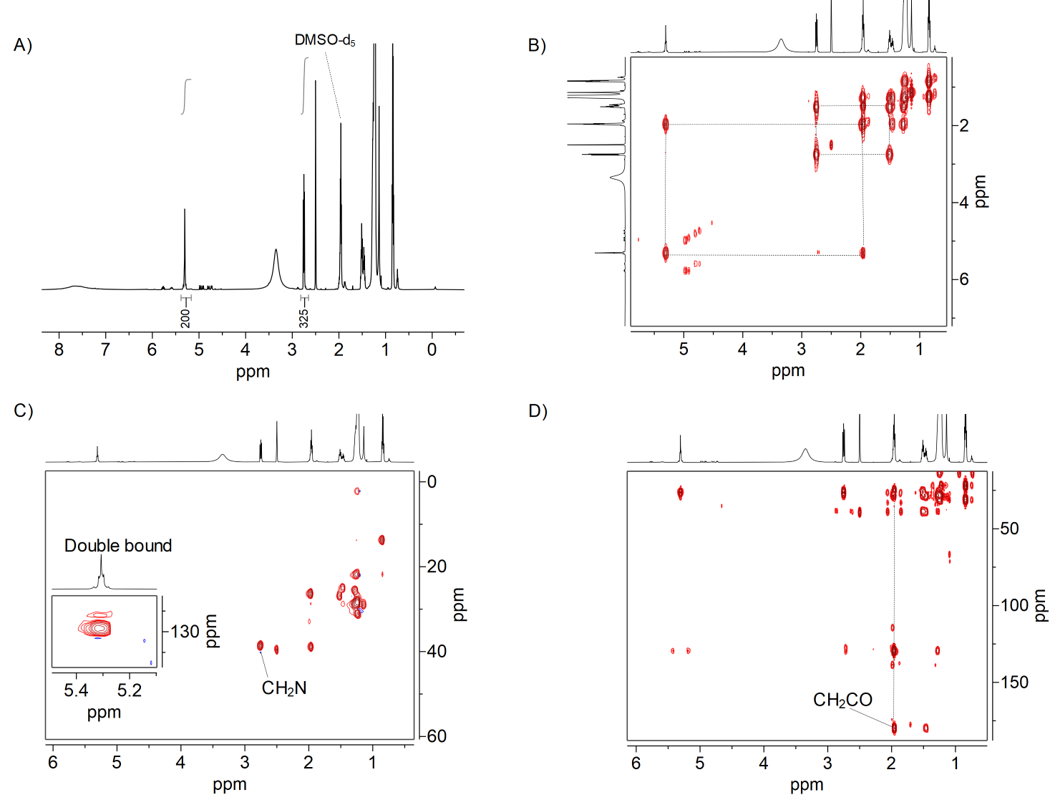
**

**Figure S11. NMR analysis on octylamine-capped nanocrystals in DMSO-d_6_.** a) Quantitative ^1^H NMR performed on a solution of free ligands in DMSO-d6, after the CsPbBr_3_ inorganic core of nanocrystals has been dissolved. The attribution of ligand-diagnostic peaks (HC=CH signal at 5.32 ppm for oleic acid and CH_2_N at 3.0 ppm for octylamine) was confirmed via ^1^H-^1^H COSY (b), ^1^H-^13^C HSQC (d), and ^1^H-^13^C HMBC spectroscopies. The relative concentration of ligands was determined by integral ratio between the CH_2_N triplet of octylamine at 2.8 ppm and the HC=CH signal at ∼5.3 ppm, attributed to oleic acid only.

# 7) Optical properties of nanocrystals in solution

Absorption and photoluminescence (PL) spectra of the nanocrystals in solution were acquired with a Cary 300 spectrophotometer and with a Cary Eclipse spectrofluorometer, respectively. The equilibrium PL and absorption spectra of the nanocrystals dispersed in toluene are shown in Figure S12a. Absolute photoluminescence quantum yields and time-dependent PL intensity decays of nanocrystals in toluene solution were measured using an Edinburgh FLS900 fluorescence spectrometer equipped with a xenon lamp, a monochromator for steady-state PL excitation, and a time correlated single photon counting unit coupled with a pulsed laser diode (λ_ex_ = 405 nm and pulse width of 50 ps) for time resolved PL. The PLQY was measured using a calibrated integrating sphere (λ_ex_ = 400 nm). All solutions were diluted to an optical density of 0.1 at the corresponding excitation wavelength, to minimize self-absorption. Time-dependent PL intensity decays for CsPbBr_3_ nanocrystals prepared with different amines are shown in Figure S12b. All decays were fitted by a double exponential decay function (Equation VIII). The decay time extracted by the fitting, together with the measured quantum yield are reported in Table S9.

$y=y_{0}+A_{1}\exp\left\{ -\frac{\left( x-x_{0} \right)}{\tau_{1}} \right\}+ A_{2}\exp\left\{ -\frac{\left( x-x_{0} \right)}{\tau_{21}} \right\}$ Eq. (VIII)

**Table S9. Optical properties of the CsPbBr_3_ nanocrystals in solution.** PL lifetime and Quantum Yield of CsPbBr_3_ nanocrystals prepared by using different amines as ligands, measured in solution.

| **Amine ligand** | **[ns]** | **[ns]** | **Quantum Yield**  **[%]** |
| --- | --- | --- | --- |
| **Oleylamine** | 3.14 ± 0.06 | 22.91 ± 0.34 | 35 ± 5 |
| **Dodecylamine** | 1.86 ± 0.02 | 7.07 ± 0.014 | 46 ± 5 |
| **Decylamine** | 1.45 ± 0.02 | 9.42 ± 0.82 | 30 ± 5 |
| **Octylamine** | 1.25 ± 0.06 | 9.48 ± 0.32 | 40 ± 5 |
| **Hexylamine** | 4.37 ± 0.07 | 12.48 ± 0.22 | 45 ± 5 |


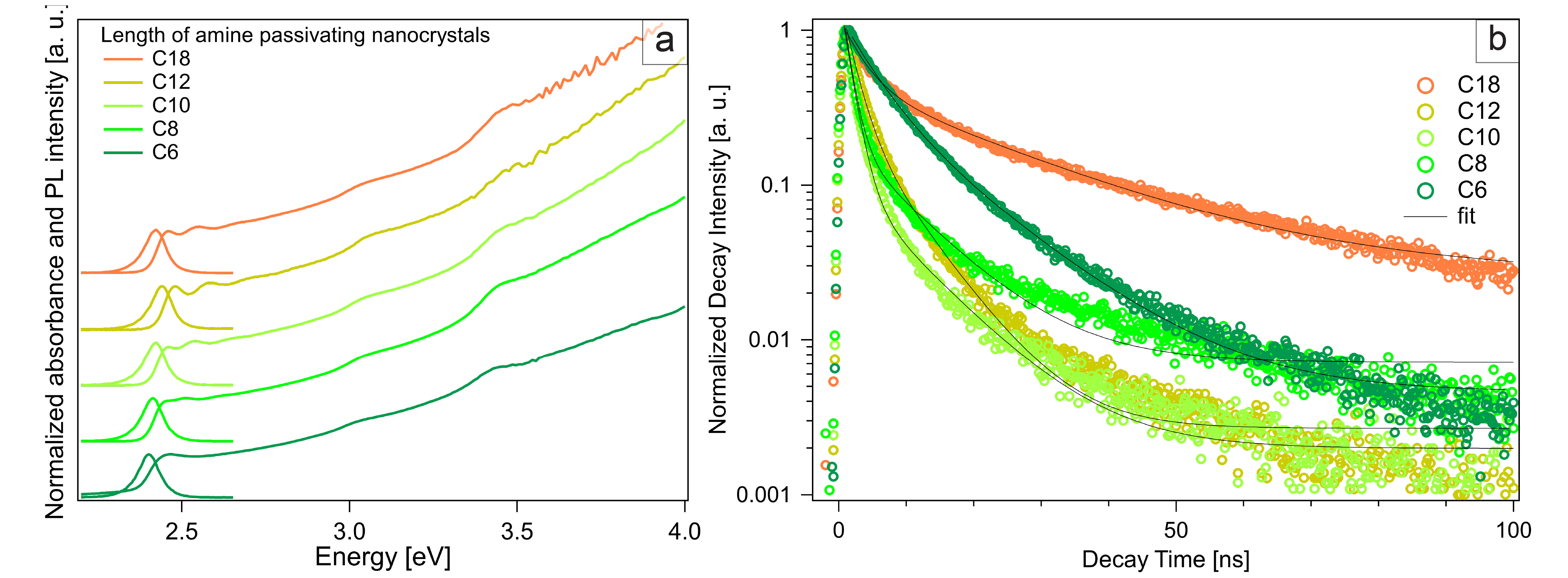


**Figure S12. Optical properties of the CsPbBr_3_ nanocrystals in solution.** (a) Room temperature PL and absorption spectra of CsPbBr_3_ nanocrystals solutions dispersed in toluene. b) PL intensity decays (colored traces) and corresponding double exponential fitting (black traces).

# 8) Transmission electron microscopy

Transmission electron microscopy (TEM) characterization of nanocrystals was performed on a JEOL JEM 1400-Plus microscope operating at 120 kV accelerating voltage. Samples for TEM were prepared by drop casting nanocrystals dispersed in toluene on top of a carbon-coated copper grid. In Figure S13a-e are shown TEM images of the nanocrystals synthesized with oleic acid and different amines. For each type of nanocrystals, approximately 15 images similar to the one in Figure S13 a-e were collected, and the size of particles visible in each image was analyzed with the software ImageJ for a total of 1500 nanocrystals per passivation time. The average nanocrystal edge lengths, calculated from the average nanoparticle areas by approximating the nanocrystal 2D projection to a square, are reported in Figure S14.


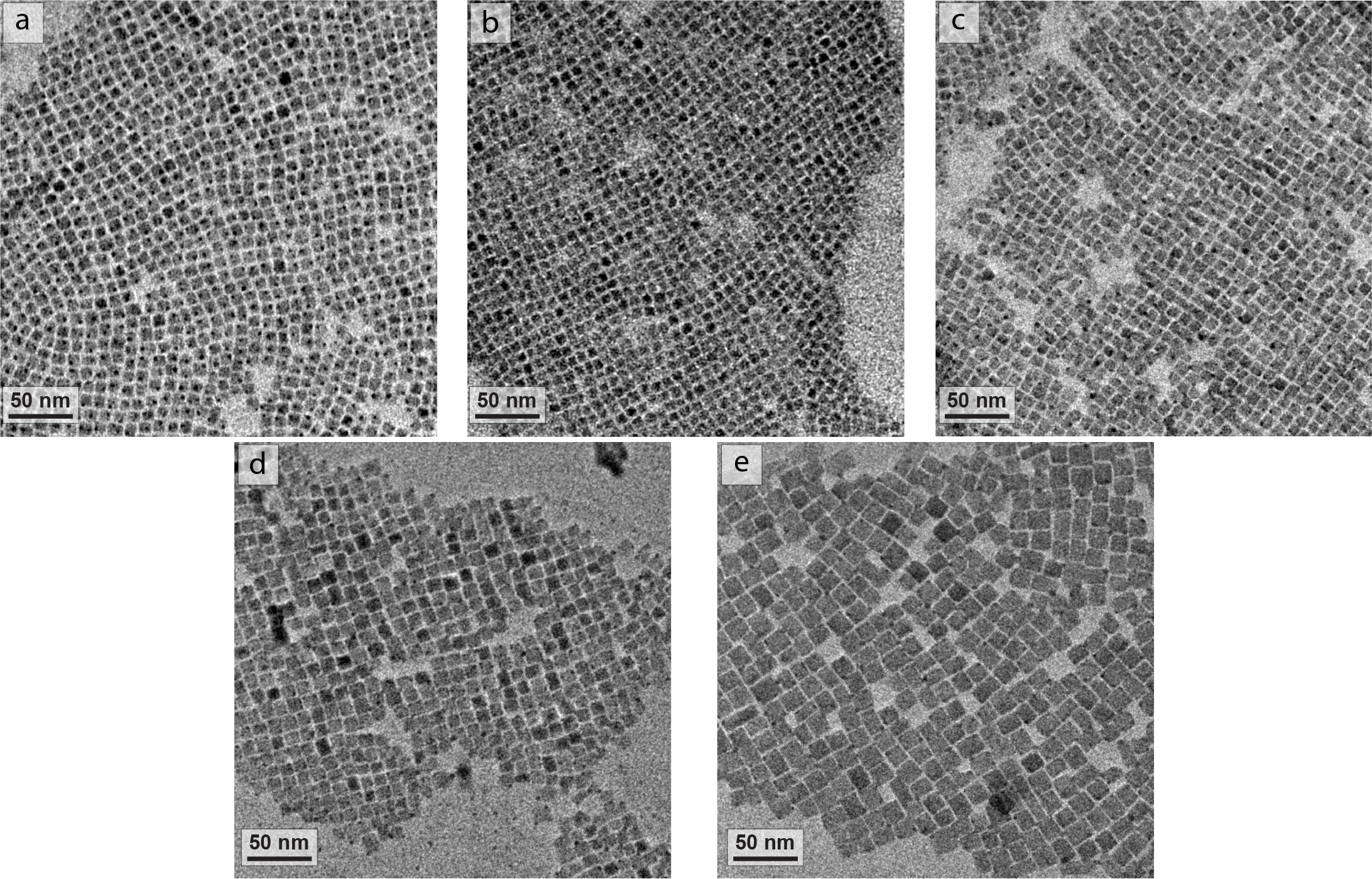


**Figure S13. CsPbBr_3_ nanocrystals.** TEM images of nanocrystals synthesized by employing as surfactants a combination oleic acid (all samples), plus oleylamine (a), dodecylamine (b), decylamine (c), octylamine (d), and hexylamine (e).

**
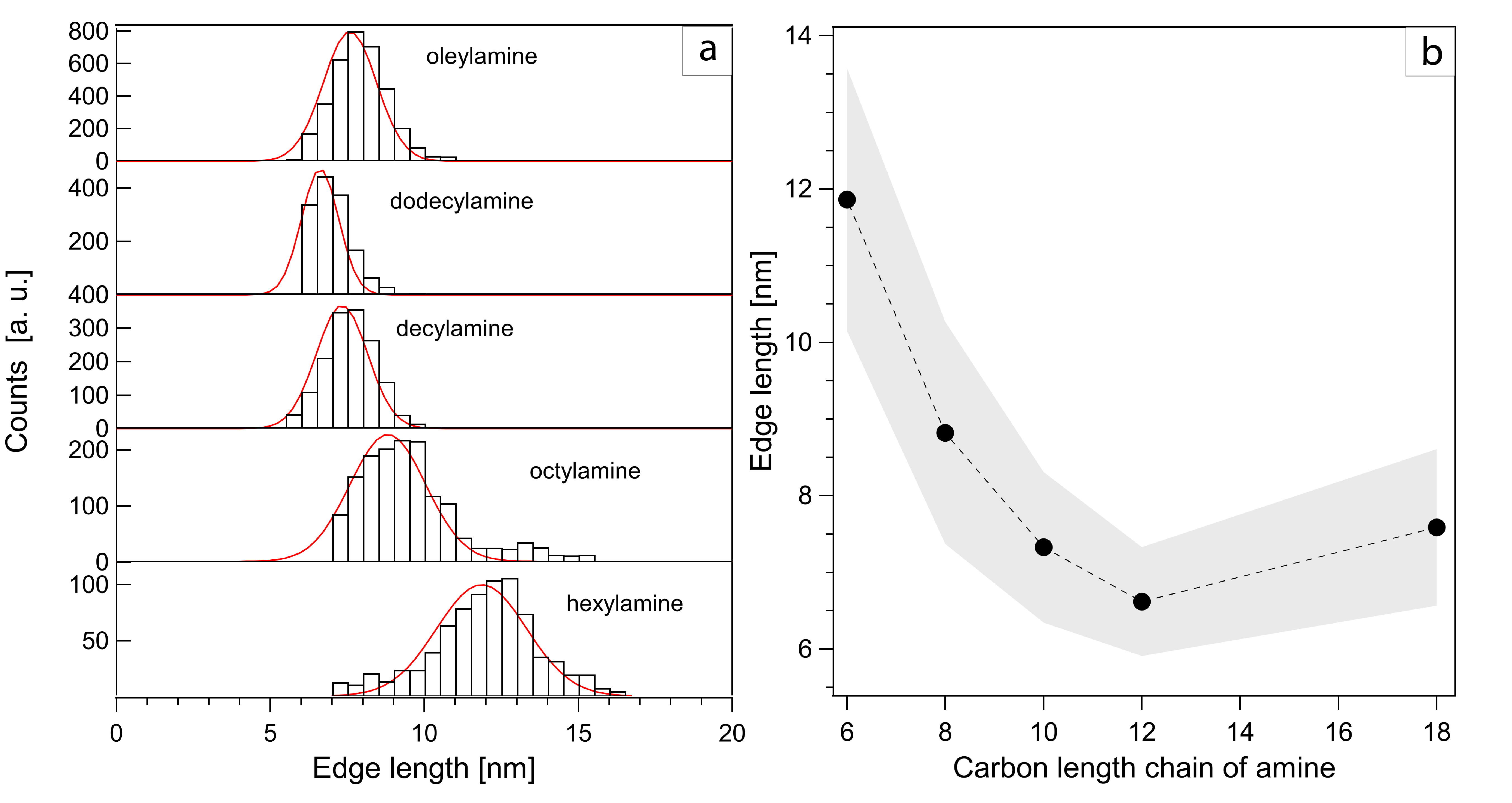
**

**Figure S14. Nanocrystals size distribution by TEM.** a) Edge length distribution and (b) edge length comparison as a function of the chain length of the amine ligand. The shaded area in (b) represents the full width at half maximum (FWHM) of the distribution.

# 9) Reproducibility of hexylamine-capped superlattices and attempts with butylamine.

Although best structural order (i.e., lowest *σ_L_*) was observed for superlattices assembled from particles capped with hexylamine, we opted to focus on octylamine-capped nanocrystals because the assembly quality of hexylammonium-capped nanocrystals was not always successful, as shown in Figure S15.


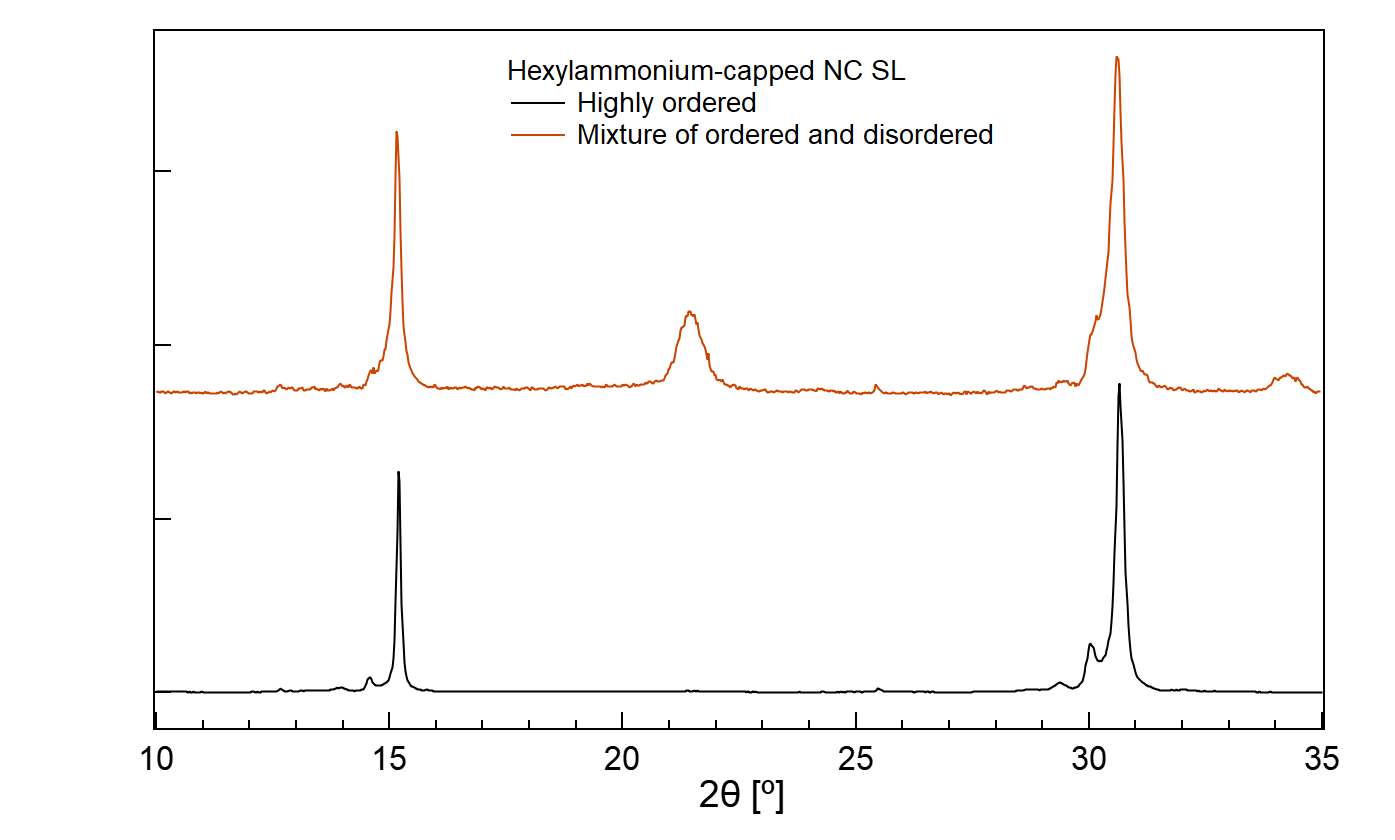


**Figure S15. Reproducibility of hexylamine-capped nanocrystal superlattices.** XRD patterns acquired in the same condition from two different sample of hexylamine superlattices, prepared as described in the experimental section in the main text. While the black pattern is typical of a successful assembly, showing only two peaks modulated with sharp fringes, the red pattern is indicates a lower assembly order (broader fringes) and the presence of non-assembled CsPbBr_3_ particles lying in random orientations (extra peak at 2θ ≈ 21.5°).


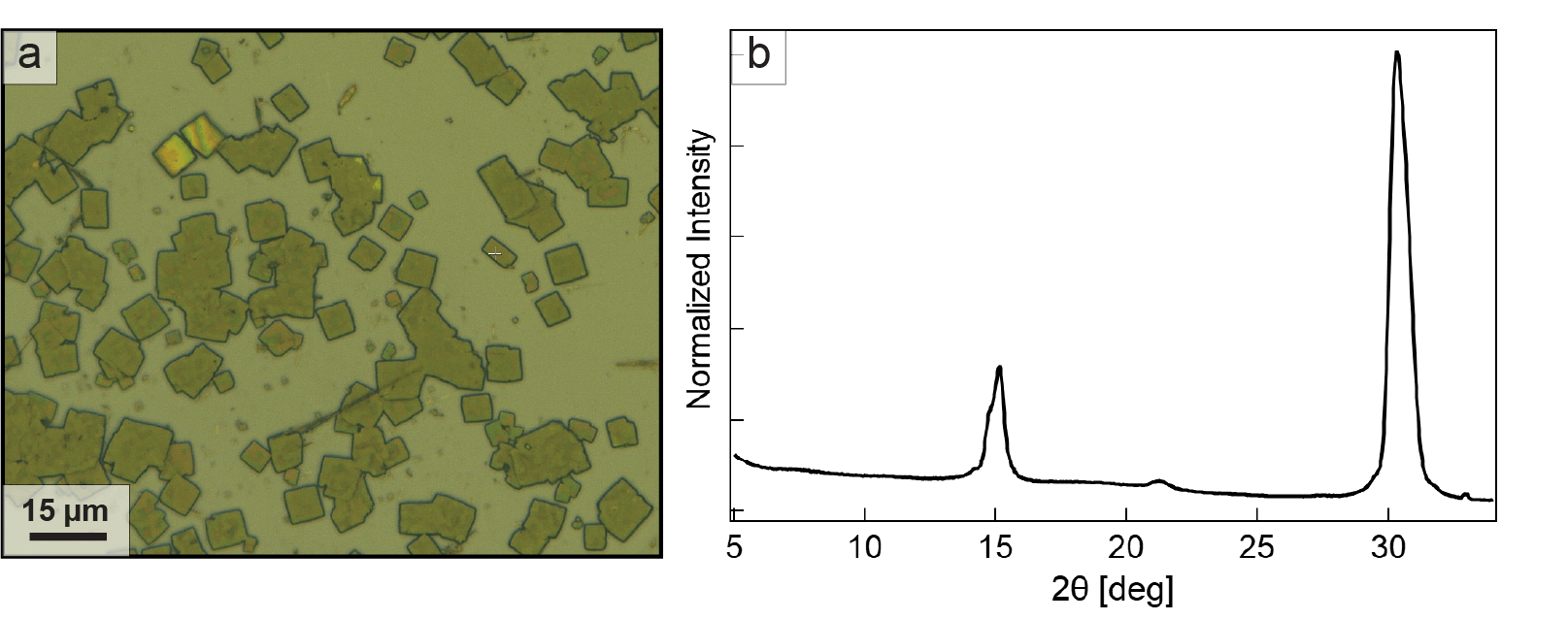


**Figure S16. Superlattices assembled from butylamine-capped nanocrystals:** a) Optical microscopy image of superlattices grown from CsPbBr_3_ nanocrystals passivated by butylamine and oleic acid. b) XRD pattern showing no multilayer diffraction interference in correspondence of Bragg reflections, which is due to the high structural disorder of the superlattices.

# 10) Cooling octylamine-capped nanocrystal superlattices in vacuum

**
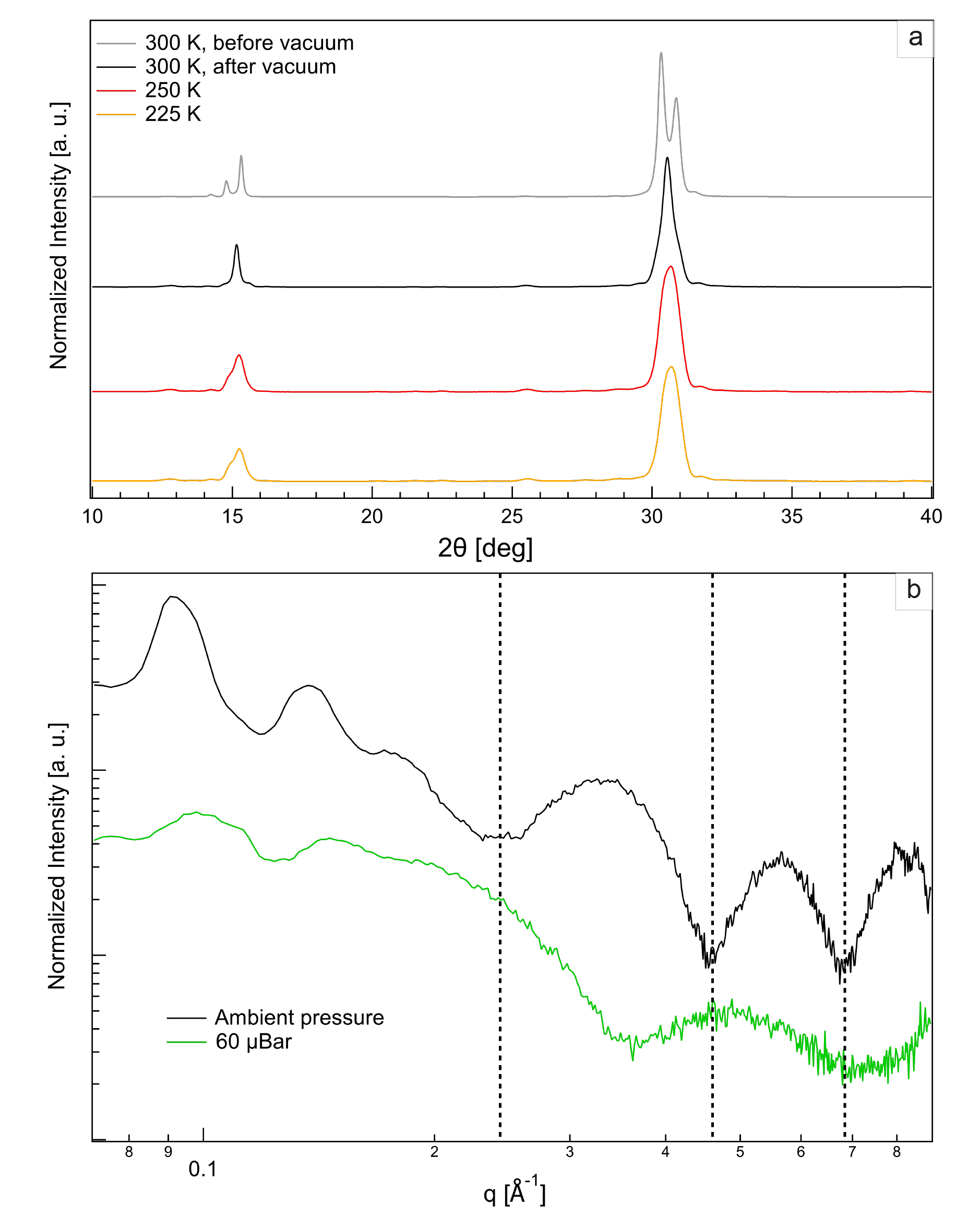
**

**Figure S17. Evolution of octylamine-capped nanocrystal superlattices upon cooling under vacuum.** a) XRD patterns acquired while cooling the sample under mild vacuum (0.5 mBar), highlighting the loss of structural coherence via the smearing of multilayer diffraction fringes. The induced disorder is sudden (at 225 K σ_L_ ≈ 1.95 Å) and irreversible (not shown). b) Small-angle XRD patterns measured at ambient pressure and after 12 hours of vacuum at 60 μBar. The variation in the periodicity of the modulated signal (0.226 Å^-1^ at ambient pressure vs 0.355 Å^-1^ under vacuum) indicates a major contraction of the interparticle distance (ΔL ≈ 10 Å). See Ref. [1] for details on the analysis of low-angle XRD signals.

# 11) Thermogravimetric Analysis

Thermo-Gravimetric analyses (TGA) were carried out on a TA Instruments TGA Q500 under inert atmosphere (N_2_, 50 mL/min) by first equilibrating the sample at 30 °C for 5 min and then heating at a constant rate of 10 °C/min up to 1000 °C. The nanocrystal solution was synthesized employing octylamine and oleic acid. Two samples were prepared by drop casting nanocrystals solutions on 0.3 0.3 cm silicon substrates. Optical absorption spectra were collected to ensure that the two solutions were equally concentrated.^[9]^ Prior to the analysis, one of the substrate was kept under vacuum ≈ 12 hours, to emulate the effects of vacuum applied in a cryostat.


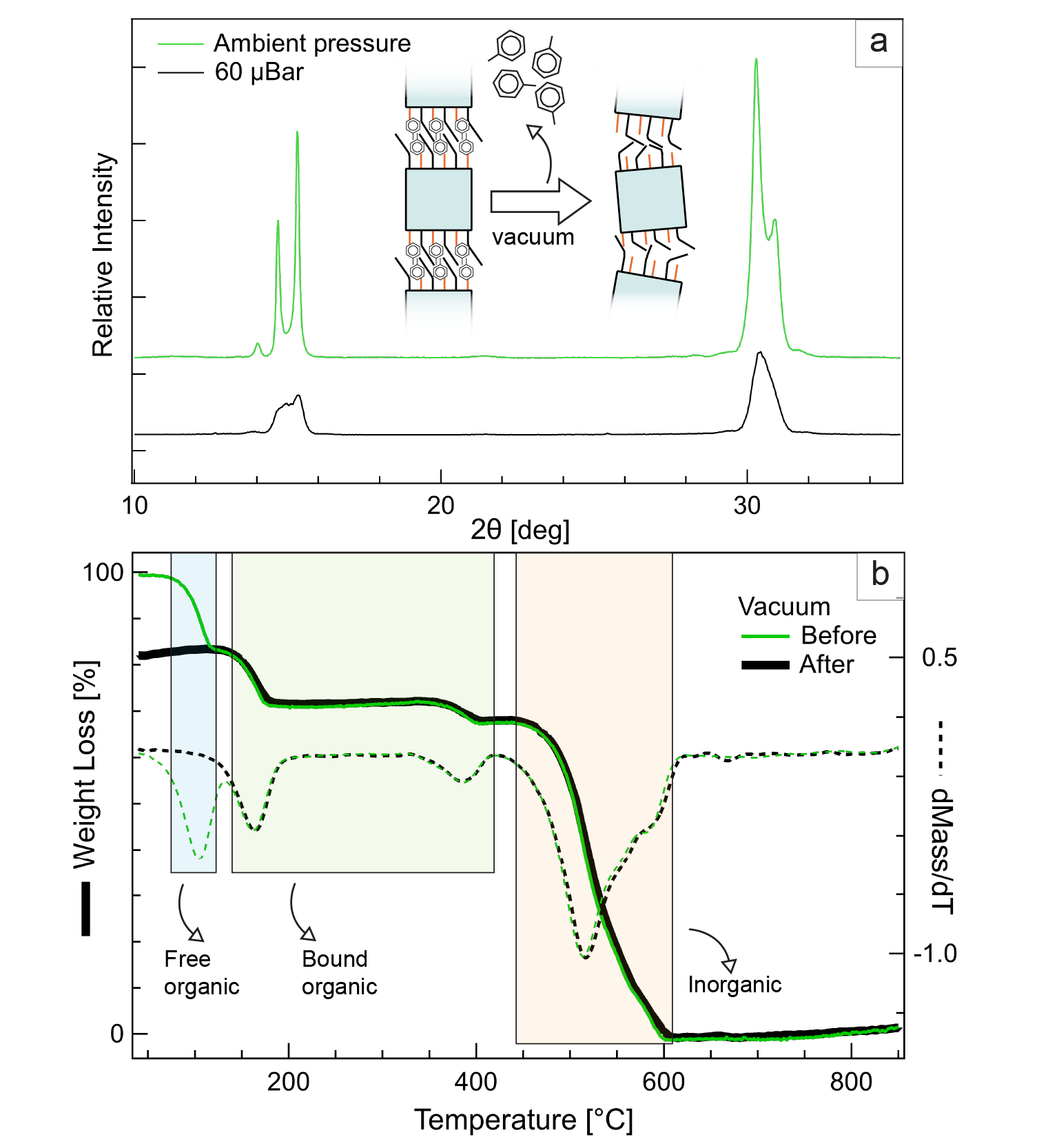


**Figure S18. Effect of vacuum on octylamine-capped nanocrystal superlattices.** (a) XRD pattern of CsPbBr_3_ nanocrystal superlattices capped with octylamine and oleic acid before (black) and after applying vacuum (green). (b) Thermogravimetric analysis performed on two samples, one pristine (green)and one pre-treated with vacuum (black). The extra weight loss seen in the green trace is attributed to free organic, like residual solvent and ligands, which is extracted by vacuum and whose sudden removal destroys the structural coherence of superlattices.

# 12) Oleylamine-capped nanocrystal superlattices measured under N_2_ atmosphere.


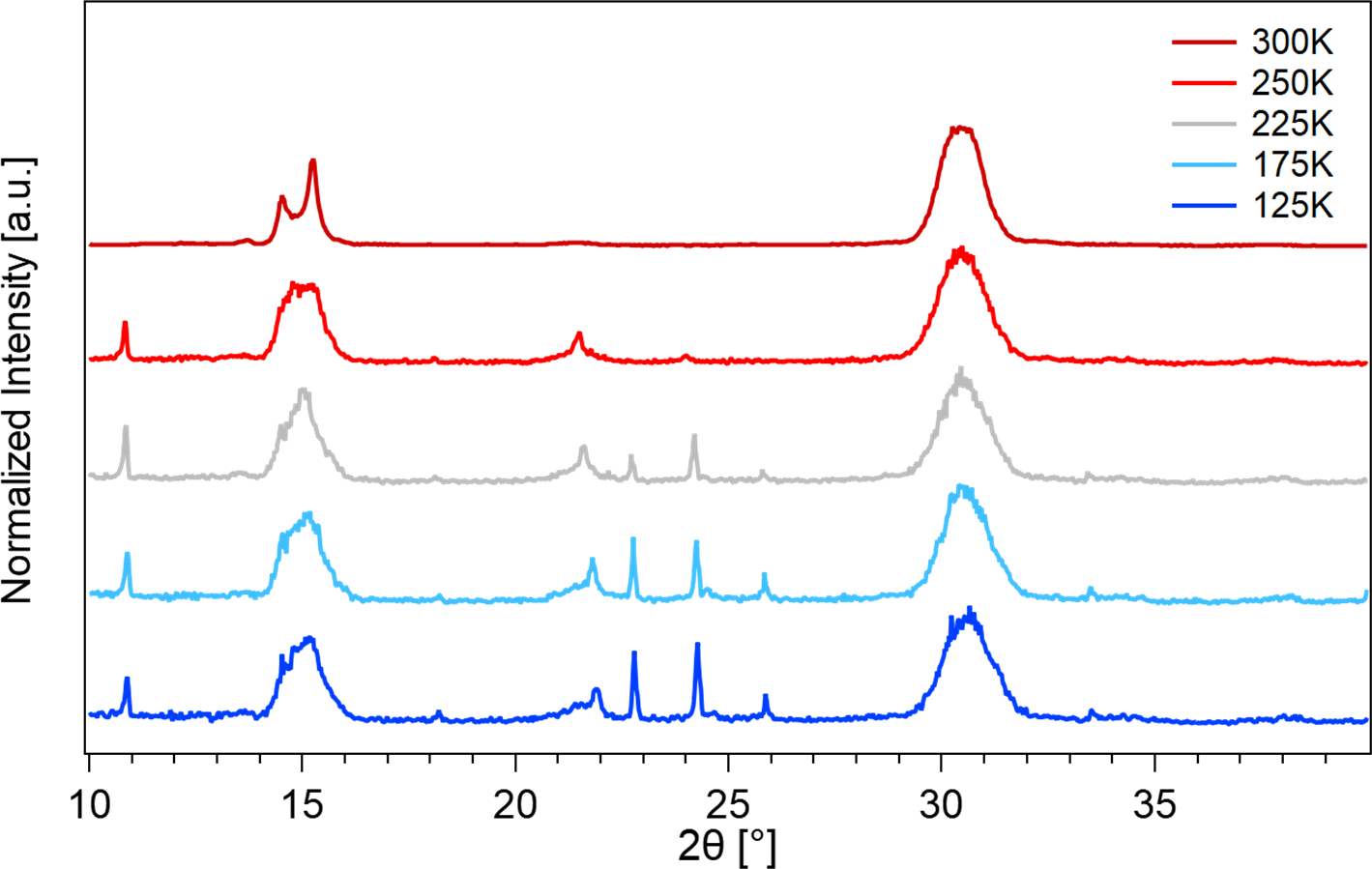


**Figure S19. Low temperature XRD patterns of oleylamine-capped nanocrystal superlattices under N_2_ atmosphere.** The observed broadening of multilayer diffraction fringes is comparable to that seen in to Figure 1 of the Main Text, where a similar sample is cooled under vacuum.

# 13) Raman spectroscopy

Raman measurements were performed with a micro-Raman spectrometer inVia (Renishaw, UK) equipped with a 633 nm laser and 1.7 mW power, and a dispersion grating with 1800 grooves/mm. The data collection and analysis was handled via the software WiRE 3.2. For detection, a ×50 long working distance microscope objectives lens was used. Spectra were collected in the 1000–1800 cm^−1^ range.


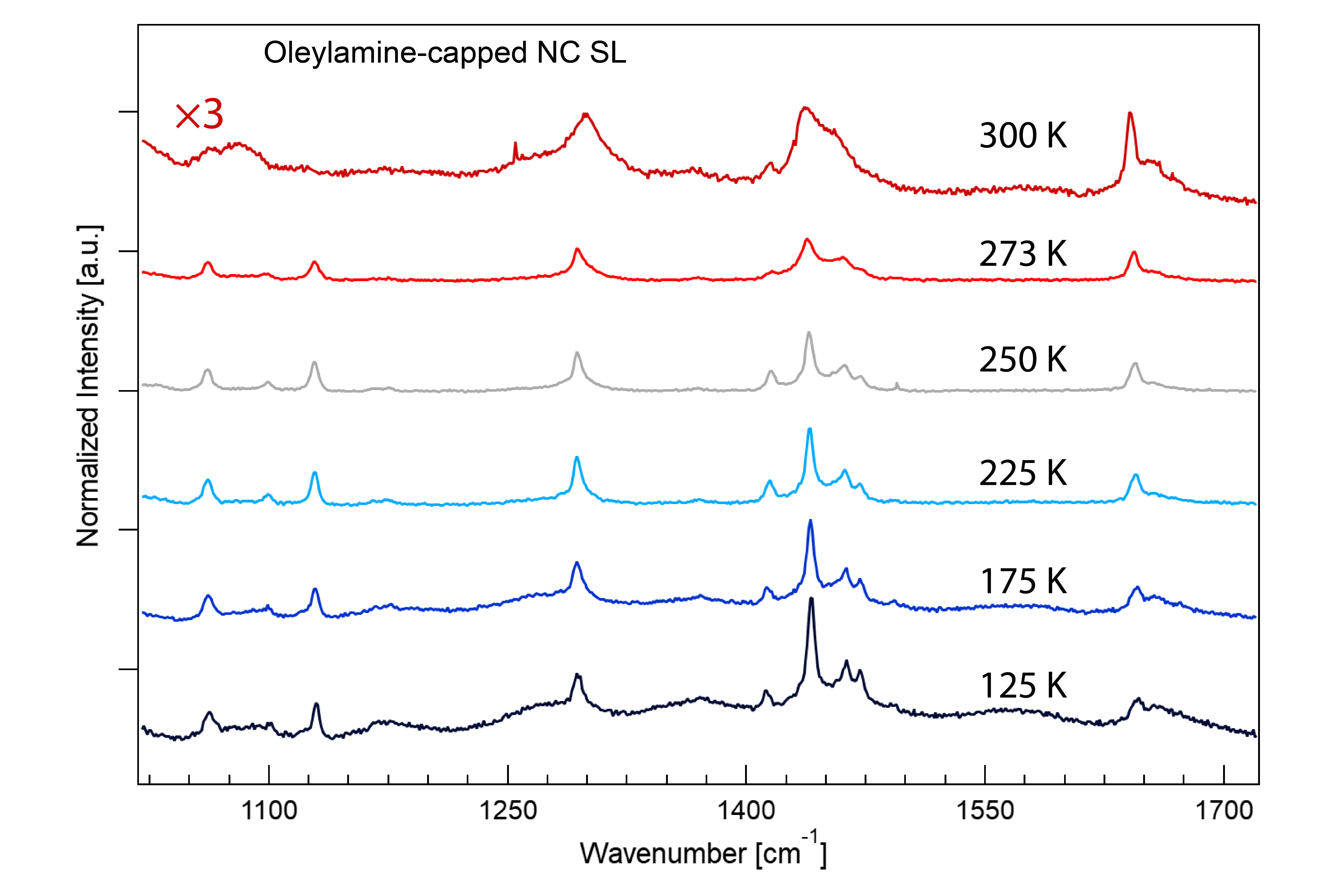


**Figure S20. Temperature-dependent Raman spectra of oleylamine-capped nanocrystal superlattices.** The spectra show a general sharpening of peaks as the temperature decreases, which is interpreted as a consequence of the solidification of ligands in between nanocrystals.


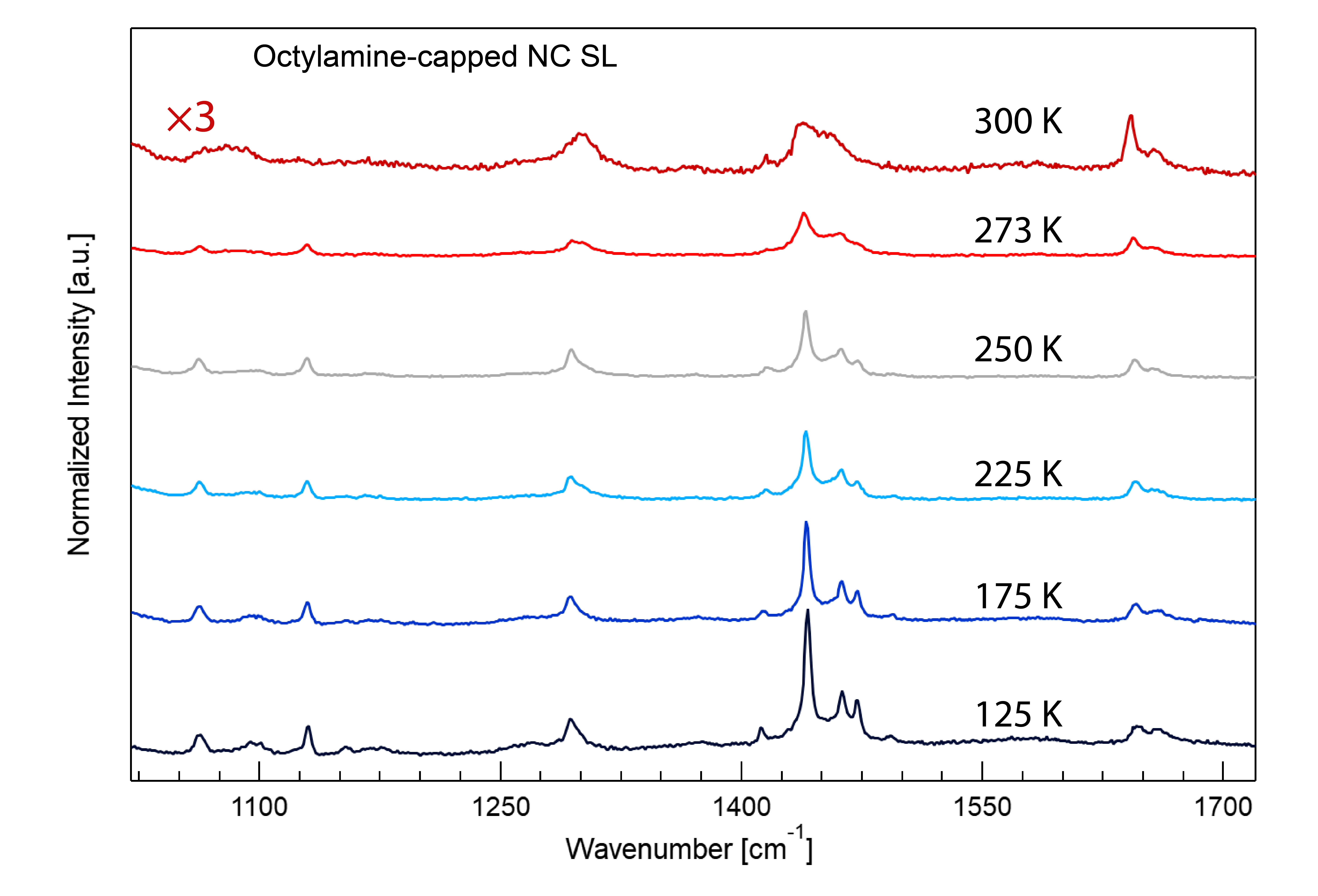


**Figure S21. Temperature-dependent Raman spectra of octylamine-capped nanocrystal superlattices.** The spectra show a general sharpening of peaks as the temperature decreases, which is interpreted as a consequence of the solidification of ligands in between nanocrystals.

**Table S10. Temperature-dependent broadening of Raman signals.** The broadening of Raman signals was measured as Full-Width at Half-Maximum (FWHM), and is here reported for several vibration modes. Reference spectral positions are indicated at 300K for all modes.

| **Temperature** | **FWHM [cm^-1^]** | | | | |
| --- | --- | --- | --- | --- | --- |
|  | **C−C**  **Antisymm. stretching**  **(1062 cm^-1^)** | **C−C**  **Symm. stretching**  **(1122 cm^-1^)** | **C−C stretching**  **(average)** | **CH_2_ scissoring (1437 cm^-1^)** | **C=C stretching (1640 cm^-1^)** |
| **300 K** | 12.4 | 16.4 | 14.4 | 11.8 | 5.3 |
| **273 K** | 5.9 | 7.1 | 6.5 | 8.0 | 5.8 |
| **250 K** | 6.0 | 6.1 | 6.1 | 5.6 | 6.3 |
| **225 K** | 6.3 | 5.7 | 6.0 | 5.2 | 6.3 |
| **175 K** | 6.5 | 6.0 | 6.3 | 5.1 | 6.4 |
| **125 K** | 7.2 | 4.9 | 6.1 | 5.1 | 6.5 |

# 14) Reversibility of rocking curves of octylamine-capped nanocrystal superlattices

**Figure S22. Influence of cooling rate on the order-disorder transition of superlattice:** a-b) θ:2θ X-ray diffraction patterns and (c-d) rocking curves of octylamine-coated nanocrystal superlattices measured in the slow (pink traces) and fast cooling (green traces) regimes at 300 K and at 125 K respectively.


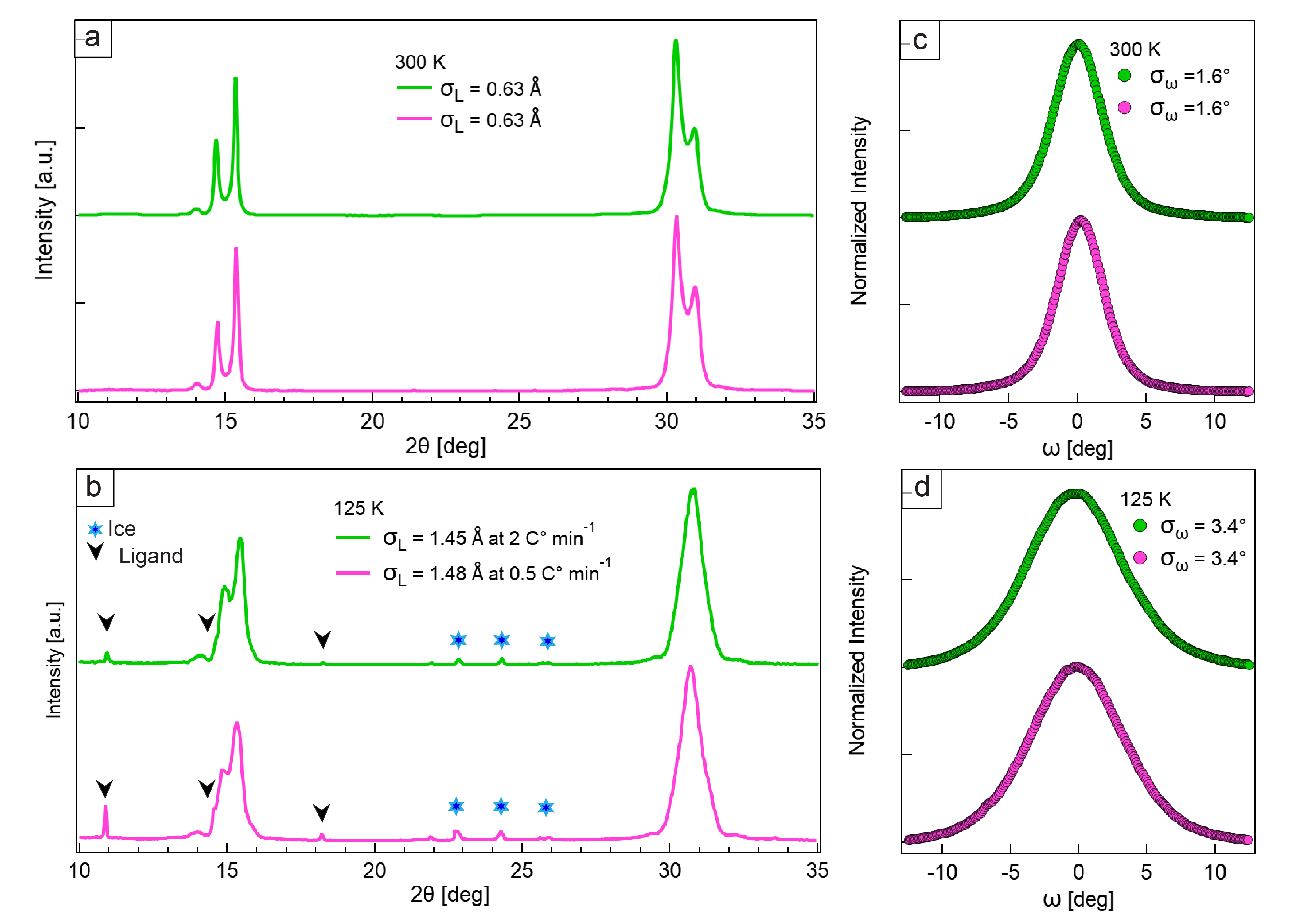


**
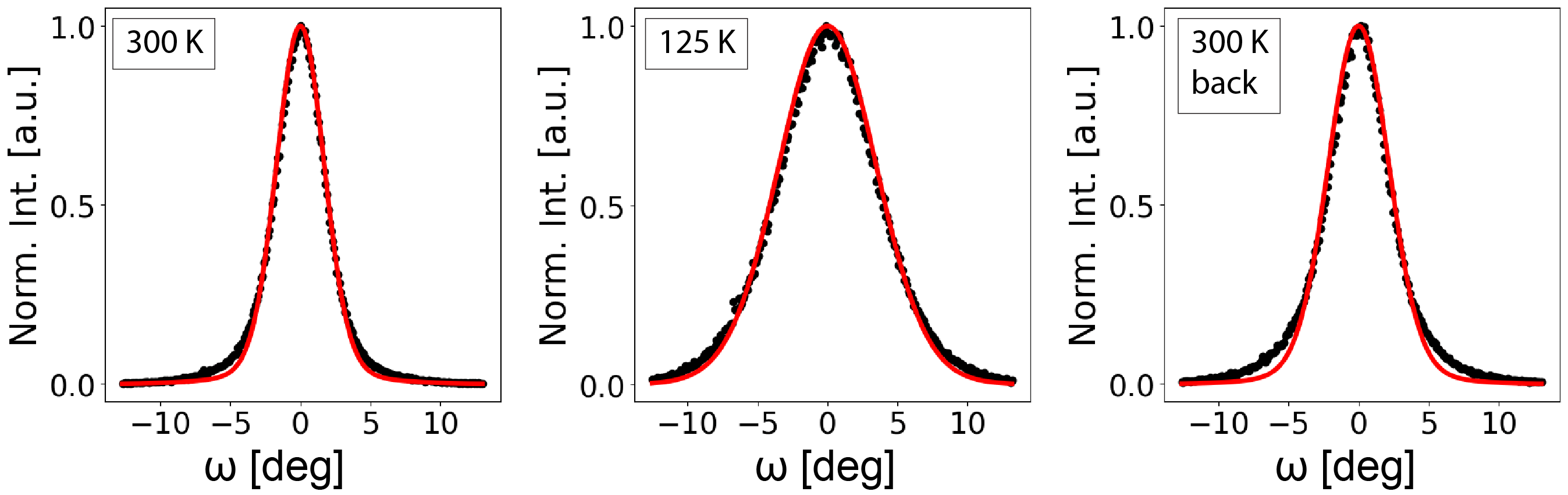
**

**Figure S23. Rocking curve reversibility.** By decreasing the duration of nitrogen exposure at cryogenic temperatures (≈ 5 hours instead of ≈ 12 hours like in Figure S6), the loss of nanocrystals orientational order could be partially mitigated, with final *σ_ω_*  = 2.0° (300 K back) almost recovering to the original value of *σ_ω_* = 1.6° (300 K), after reaching a maximum of 3.4° at 125 K.

# 15) Characterization of disordered nanocrystals film

The XRD pattern of *C_8_*-capped nanocrystals embedded in polystyrene (Figure S24a) shows peaks compatible with the crystal structure of CsPbBr_3_ (blue reference), which are crucially not modulated by multilayer interference, confirming the higher disorder of nanocrystals embedded in polystyrene. Examination by TEM highlights the co-existence of well-dispersed nanocrystals plus small, partially self-assembled domains, which however are likely more disordered than in superlattices (Figure S24b, c).

**
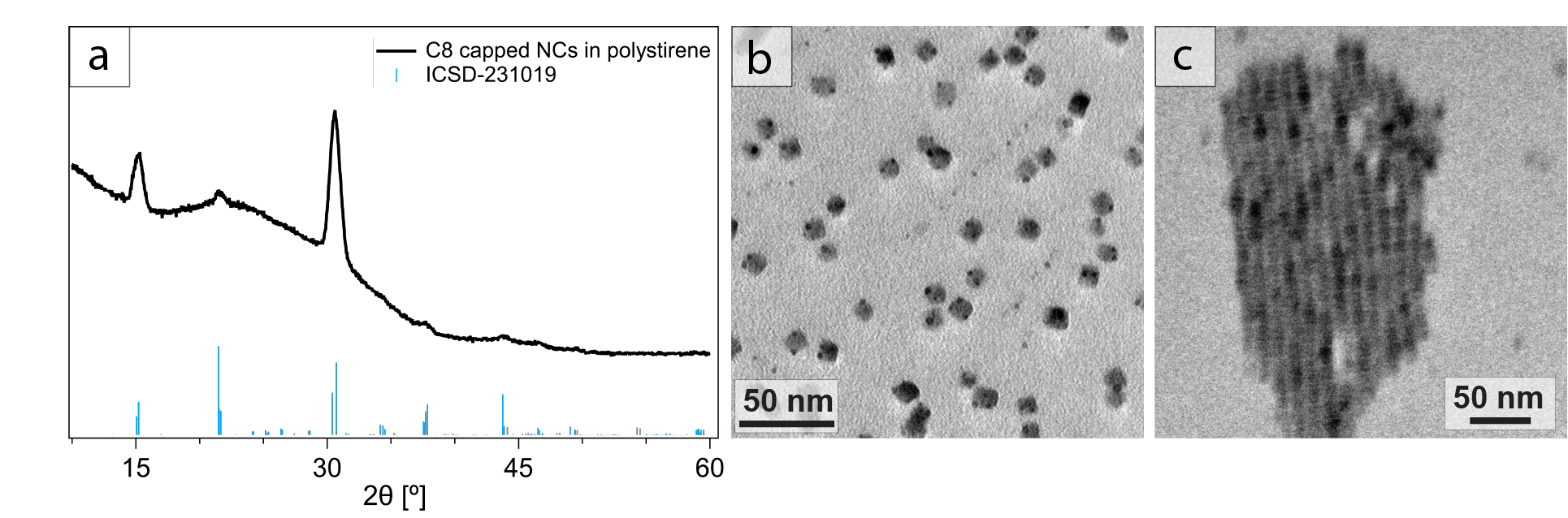
Figure S24. Characterization of composite polystyrene-nanocrystal films.** a) XRD pattern of a disordered *C_8_*-capped nanocrystal film obtained by embedding particles in a polystyrene matrix. b), c) TEM images of a diluted film prepared on a TEM. The blurriness is due to the presence of polystyrene.


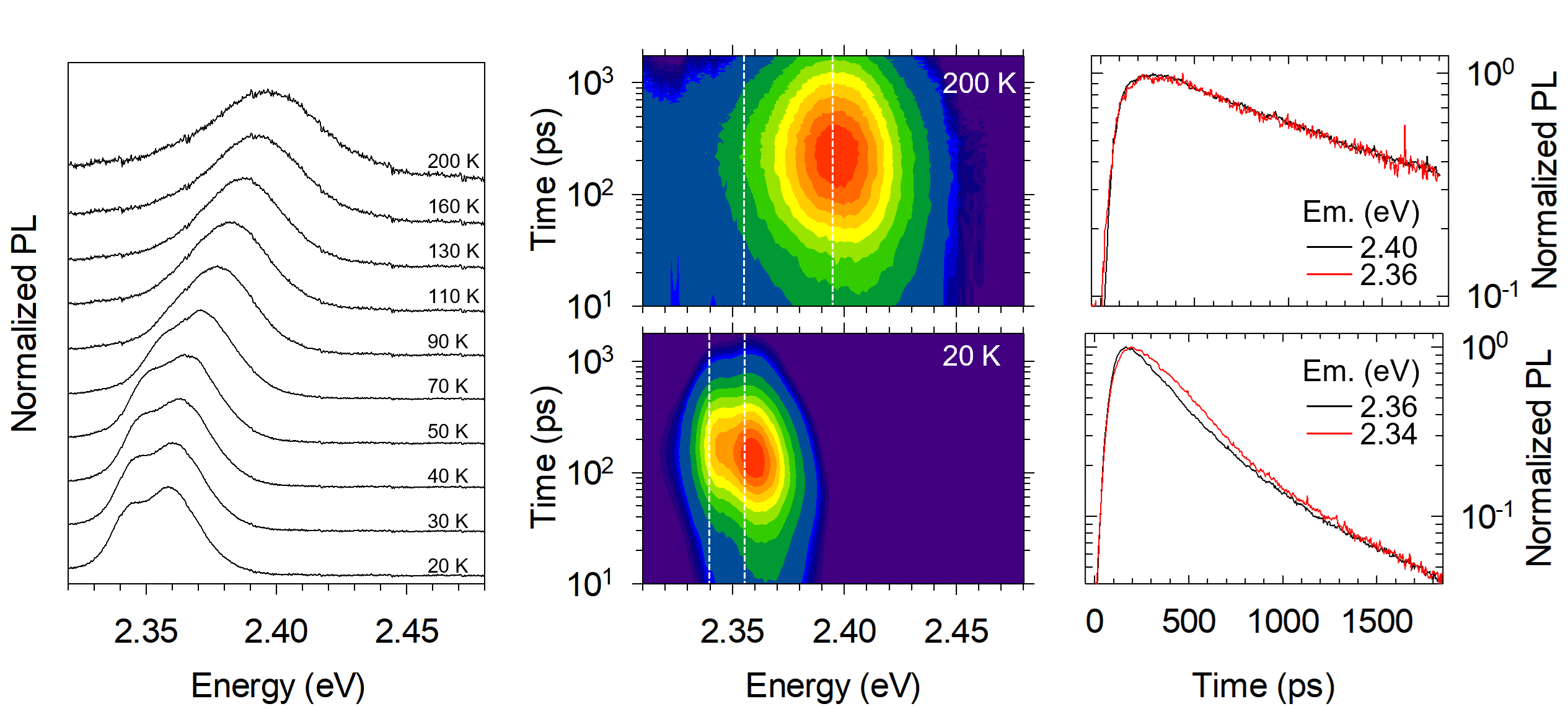


**Figure S25. PL properties of spin-cast film of C_8_-capped nanocrystals on silica.** a) PL spectra at decreasing temperature from 200K to 20K. b) Corresponding contour plots of the spectrally resolved time dynamics at 200K and 20K together with c) the respective decay curves at the indicated energy values. Measurements acquired at 3 nJ/cm^2^ excitation fluence.

# 16) Optical properties of C_8_ and C_18_ superlattices.

Figure S26 shows the PLQY (photoluminescence quantum yield) values and corresponding lifetimes of C_8_-capped superlattices between 200K and 20K (Figure 6c,f of the main text). PL lifetime (extracted in correspondence of the PL maximum) decreases significantly on cooling. We believe this is due to the combined effects of a bright state exciton substructure and the activation of the giant oscillator strength due to delocalization of the transition dipole between multiple unit cells within individual nanocrystals. The PLQY simultaneously increases and saturates at about 100K due to the suppression of trapping losses typical of CsPbBr_3_ NCs.

**Figure S26.** PL decay traces, effective lifetimes (extracted as the time after which the intensity has dropped by a factor e) and PLQY values for C_8_-capped superlattices as a function of temperature.

**Figure S27.** **PL properties of C_18_-capped nanocrystal superlattices.** a) PL spectra at 200 K (black trace) and 20 K (blue trace). Panel b) and c) report, respectively, the corresponding contour plots of the spectrally resolved PL kinetics and representative decay curves at representative energies. Measurements acquired at 3 nJ/cm^2^ excitation fluence.


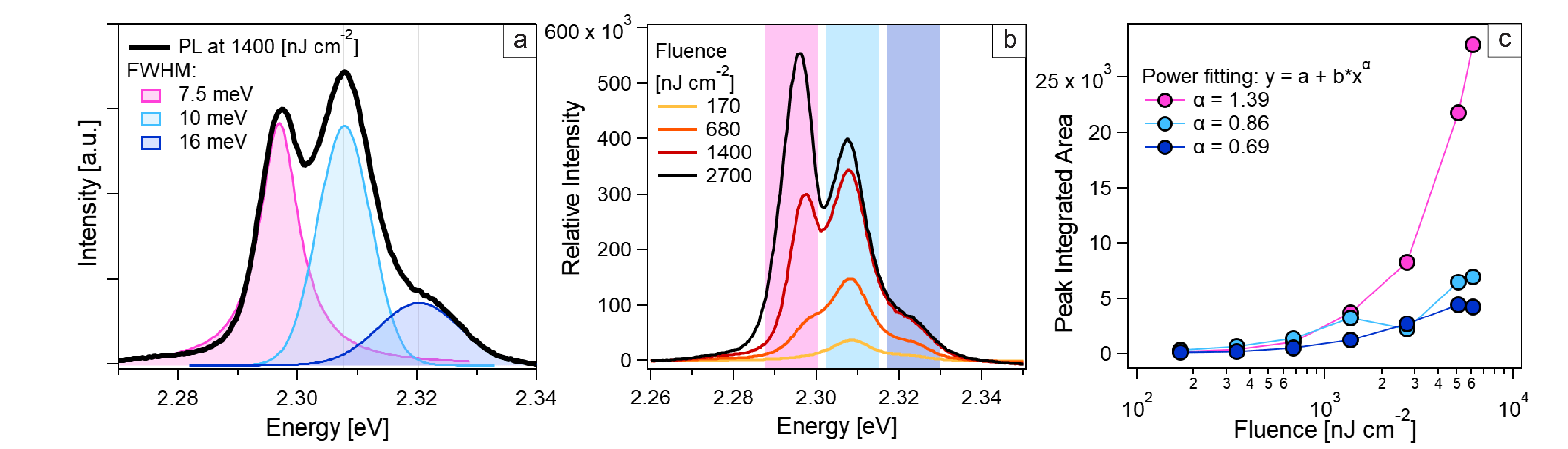


**Figure S28. High fluence PL spectra of C_8_-capped nanocrystal superlattices.** a) PL spectrum measured at 1400 nJ cm^-2^, b) fluence-dependent PL spectra and c) corresponding integrated emission intensities measured at 4 K.

# References

[1] C. A. López, C. Abia, M. C. Alvarez-Galván, B. K. Hong, M. V. Martínez-Huerta, F. Serrano-Sánchez, F. Carrascoso, A. Castellanos-Gómez, M. T. Fernández-Dĺaz, J. A. Alonso, *ACS Omega* **2020**, *5*, 5931.

[2] S. Toso, D. Baranov, D. Altamura, F. Scattarella, J. Dahl, X. Wang, S. Marras, A. P. Alivisatos, A. Singer, C. Giannini, L. Manna, *ACS Nano* **2021**, *15*, 6243.

[3] S. Toso, D. Baranov, C. Giannini, L. Manna, *ACS Nano* **2021**, *15*, 20341.

[4] S. Toso, D. Baranov, U. Filippi, C. Giannini, L. Manna, *Acc Chem Res* **2023**, *56*, 66.

[5] A. Travesset, *ACS Nano* **2017**, *11*, 5375.

[6] J. Hallstrom, I. Cherniukh, X. Zha, M. V. Kovalenko, A. Travesset, *ACS Nano* **2023**, *17*, 7219.

[7] X. Zha, A. Travesset, *Journal of Chemical Physics* **2020**, *152*, DOI 10.1063/1.5132747.

[8] P. S. C. Wu, G. Otting, *Journal of Magnetic Resonance* **2005**, *176*, 115.

[9] J. Maes, L. Balcaen, E. Drijvers, Q. Zhao, J. De Roo, A. Vantomme, F. Vanhaecke, P. Geiregat, Z. Hens, *Journal of Physical Chemistry Letters* **2018**, *9*, 3093.
